# MicroRNA Expression Profiles of Whole Blood in Lung Adenocarcinoma

**DOI:** 10.1371/journal.pone.0046045

**Published:** 2012-09-28

**Authors:** Santosh K. Patnaik, Sai Yendamuri, Eric Kannisto, John C. Kucharczuk, Sunil Singhal, Anil Vachani

**Affiliations:** 1 Department of Thoracic Surgery, Roswell Park Cancer Institute, Buffalo, New York, United States of America; 2 Division of Thoracic Surgery, University of Pennsylvania, Philadelphia, Pennsylvania, United States of America; 3 Pulmonary, Allergy and Critical Care Division, University of Pennsylvania, Abramson Research Center, Philadelphia, Pennsylvania, United States of America; Pontificia Universidad Catolica de Chile, Chile

## Abstract

The association of lung cancer with changes in microRNAs in plasma shown in multiple studies suggests a utility for circulating microRNA biomarkers in non-invasive detection of the disease. We examined if presence of lung cancer is reflected in whole blood microRNA expression as well, possibly because of a systemic response. Locked nucleic acid microarrays were used to quantify the global expression of microRNAs in whole blood of 22 patients with lung adenocarcinoma and 23 controls, ten of whom had a radiographically detected non-cancerous lung nodule and the other 13 were at high risk for developing lung cancer because of a smoking history of >20 pack-years. Cases and controls differed significantly for age with a mean difference of 10.7 years, but not for gender, race, smoking history, blood hemoglobin, platelet count, or white blood cell count. Of 1282 quantified human microRNAs, 395 (31%) were identified as expressed in the study’s subjects, with 96 (24%) differentially expressed between cases and controls. Classification analyses of microRNA expression data were performed using linear kernel support vector machines (SVM) and top-scoring pairs (TSP) methods, and classifiers to identify presence of lung adenocarcinoma were internally cross-validated. In leave-one-out cross-validation, the TSP classifiers had sensitivity and specificity of 91% and 100%, respectively. The values with SVM were both 91%. In a Monte Carlo cross-validation, average sensitivity and specificity values were 86% and 97%, respectively, with TSP, and 88% and 89%, respectively, with SVM. MicroRNAs *miR-190b*, *miR-630, miR-942*, and *miR-1284* were the most frequent constituents of the classifiers generated during the analyses. These results suggest that whole blood microRNA expression profiles can be used to distinguish lung cancer cases from clinically relevant controls. Further studies are needed to validate this observation, including in non-adenocarcinomatous lung cancers, and to clarify upon the confounding effect of age.

## Introduction

Lung cancer contributes to more cancer deaths annually in the United States than colorectal, breast and prostate cancers combined [1]. Recent advances in the clinical management of lung cancer have led to only small improvements in overall survival for the disease, in part because a majority of the cases are identified only after the cancer has advanced to a more malignant stage. Screening of individuals at a higher risk of developing lung cancer to diagnose the disease at an earlier stage therefore has the potential to improve clinical outcome of the disease. This is supported by results of the National Lung Cancer Screening Trial that show an approximately 20% improvement in lung cancer-related mortality with annual low-dose computerized tomographic screening [2]. However, in the trial, 96% of the pulmonary abnormalities seen were benign lesions. Periodic radiological tests for screening may also expose individuals to a significant level of radiation, the impact of which is unknown but possibly harmful. In routine clinical practice, the incidence of pulmonary nodules detected in chest radiography ranges from 0.09% to 0.2% and is higher in more advanced radiological examinations [3], [4]. The chance of such a nodule being malignant varies widely from 1% to 70% [3], [5], and depends on a number of factors such as the size of the nodule and the clinical setting. The detection of a lung nodule in a radiological examination can thus not only cause patient anxiety but lead to tests such as positron emission tomography and biopsy that can be invasive, often expensive, and likely of no benefit for a large proportion of individuals. A non-invasive (e.g., blood-based) biomarker assay for the presence of lung cancer that can complement or replace radiological examination during screening or routine clinical visits can therefore be useful in identifying subjects that are most likely to have a malignant lesion in the lung that requires further investigation.

At least 18 non-invasive, blood-based studies have examined microRNA expression profiles to identify microRNA biomarkers for diagnosis of lung cancer. Most of them have quantified microRNAs in the non-cellular serum (e.g., [6], [7], [8]) or plasma (e.g., [9], [10], [11]) fractions of blood. Although all these studies, except one using plasma microRNA expression [12], have shown promising results, the use of serum or plasma RNA for microRNA biomarker discovery has some limitations. The yield of RNA from human serum and plasma is estimated to be in the range of 2.5–120 ng/ml (e.g., [13], [14], [15]) and this limits unbiased biomarker discovery by affecting reliable and accurate detectability of microRNAs in global expression profiling assays. Isolation of serum or plasma also involves additional steps, and microRNA expression patterns can be sensitive to minor variations during these processing steps (e.g., [12], [16]). Furthermore, because cellular microRNAs are overwhelmingly more in amount than extracellular ones, even a very small degree of contamination of the isolated serum or plasma samples with blood cells significantly alters their microRNA expression profiles (e.g., [16], [17]).

The mechanistic basis for the alterations in serum or plasma microRNAs consequent to the presence of lung cancer is not clear. It could be that tumors themselves release microRNAs into circulation, as is suggested by the findings of some studies (e.g., [18], [19]). However, it is unlikely that it is so for at least a majority of the altered microRNAs [20]. It is believed that microRNAs are released into blood circulation by all cells of the body [21] and not just tumors which typically constitute only a very small fraction of the body’s cellular mass. No microRNA is exclusively expressed by cancer cells, and the fold-changes in microRNA expression levels that occur in cancer tissues relative to normal ones are usually very modest (e.g., [22]). It is therefore possible that the changes in microRNA expression seen in serum or plasma reflect the body’s systemic response to the presence of cancer, including changes in microRNA expression in circulating blood cells [17]. Such a response may be exhibited in whole blood microRNA expression. Indeed, a number of recent studies have shown changes in microRNA expression profiles of peripheral whole blood in patients with various malignancies, such as brain [23], breast [24], ovary [25], and pancreas [26], as well as in non-malignant diseases [27], [28], [29].

The goal of this study was to examine the potential of whole blood microRNA profiling to distinguish patients with lung adenocarcinoma, which accounts for about a half of lung cancer, from clinically relevant controls. Whole blood mRNA expression changes have been associated with presence of lung cancer [30], and four studies so far have identified whole blood microRNA biomarkers associated with the presence of lung cancer [31], [32], [33], [34]. Three of these four studies were published while the work described here was in progress.

## Materials and Methods

### Ethics Statement

This study was approved by the Institutional Review Board of University of Pennsylvania (study identification number 806390).

### Study Population and Blood Collection

Study participants included 22 patients with lung adenocarcinoma (cases) and 23 patients without lung cancer (controls) who were evaluated at the University of Pennsylvania between November 2007 and October 2010. Peripheral blood (2.5 ml) was collected from the participants during clinical visits in a PAXgene™ Blood RNA tube (Qiagen®, Valencia, CA), which was then frozen at −20°C within 2 hours and then transferred to −80°C within a day for long-term storage. None of the case subjects received any treatment for cancer prior to blood collection. Ten controls underwent surgery for a suspicious lung nodule or mass that on pathological evaluation later was found to be benign. The remaining 13 controls were older than 50 years with a smoking history of >20 pack-years. White blood cell (WBC) and platelet counts, and blood hemoglobin values at time-points closest to the time of blood collection for RNA isolation were collated from medical records. These were identified before surgery in all but one case for which the values were obtained immediately after surgery. For controls, blood counts and hemoglobin values could be obtained for 17 (74%) subjects; for six of them, the values were determined >90 days before blood had been collected for RNA isolation.

### Isolation of RNA from Blood

Total RNA including small RNA was isolated from blood collected in PAXgene™ Blood RNA tubes using the PAXgene™ Blood miRNA kit (Qiagen®) as per the protocol supplied by the manufacturer. RNA was collected in 80 µl of the BR5 buffer provided with the kit. Concentration and quality of RNA was assessed by absorbance spectrometry on NanoDrop™ 2000 (Thermo®, Waltham, MA) and imaging of ethidium bromide-stained RNA electrophoresed on an agarose gel.

### MicroRNA Quantification by Locked Nucleic Acid Microarray

This work was performed as a commercial service by Exiqon® (Vedbaek, Denmark). The miRCURY™ microRNA Power Labeling kit (Exiqon®) was used to 3′- or 5′-end label 0.5 µg of a sample or a human ‘universal reference’ total RNA (Ambion®, Austin, TX; product number AM6000) with the Cy3-like Hy3™ or the Cy5-like Hy5™ (Exiqon®) dye, respectively, before they were co-hybridized overnight to 5th generation miRCURY™ locked nucleic acid microarrays (Exiqon®) [35]. After washing, microarrays were scanned and analyzed using ImaGene® software (version 9; BioDiscovery®, Los Angeles, CA). Manual and automated examinations of the scans and analyses of microarray signals for 52 spiked-in synthetic, small RNAs showed that all labeling reactions and hybridizations were of good quality. The arrays had more than 1890 locked nucleic acid probes for multiple RNAs of human, mouse, rat, and some viruses printed in quadruplicate on randomly distributed spots of 105 µm diameter and 250 µm inter-spot distance. A total of 1305 probes on the arrays targeted 1282 human microRNAs, including 376 proprietary ones (miRPlus™, Exiqon®), and 23 non-microRNA human small RNAs of <200 nucleotides, including the *5S* ribosomal RNA and the two *RNU6* small nucleolar *U6* RNAs. Except for *RNU6-1* (*U6A*), every RNA was recognized by only one of the 1305 probes. Only eight of the 1268 probes against human microRNAs and one of the 24 against human non-microRNAs recognized more than one species of RNA. In this study, the multiple RNAs recognized by such probes are enumerated individually even though the analyses of microarray signals considered each probe and not each microRNA as a separate variable. Raw and pre-processed microarray data are available online in the Gene Expression Omnibus database [36] with accession number GSE27486.

### Pre-processing of Microarray Data

Hy3™ and Hy5™ signal values from the 45 hybridizations were processed together using the limma [37] Bioconductor package (version 3.6.9) and custom code in R (version 2.12). Raw values were corrected for background noise using the convolution model-based normexp method [38] with an *offset* of 10, and then normalized, first within array by the global loess regression method [39] with a *span* of 1/3, and then between arrays by the limma *Rquantile* method to achieve identical distributions of Hy5™ values among all hybridizations. Microarray signal values were then identified as summarized Hy3™ values which were the means of values from the multiple probe-spots when the maximum was <1.5x of the minimum, or the medians if otherwise. At this point, data from probes that did not recognize human RNAs was removed. RNAs recognized by probes for which the microarray signal values were >3x that of probe-less empty microarray spots in at least a quarter of the 45 hybridizations were considered as expressed. There were 548 probe-less empty spots on each array, and the mean and range of signal values from all such spots on all 45 arrays were 11.0 and 8.6–12.4, respectively. Microarray signal values for the expressed RNAs were used for further analyses.

### Analyses of Microarray Signals

Differential expression analyses were performed using empirical Bayes-moderated t-statistics with the limma Bioconductor package. Differentially expressed RNAs were identified as those with false discovery rates of <5% as per the Benjamini-Hochberg method. Classification analyses of microarray signals for expressed microRNAs were done in R using the CMA [40] Bioconductor package (version 1.8.1) for the support vector machines (SVM; linear kernel) method, and the tspair [41] Bioconductor package (version 1.8) for the top-scoring pairs (TSP) method [42]. Internal validation was performed using the leave-one-out and Monte Carlo cross-validation methods (LOOCV and MCCV, respectively). In LOOCV, training sets of 44 samples consisted of all but the one sample that formed the test set. In MCCV, the 45 samples of the study were randomly assigned to training and test sets of 36 and 9 samples, respectively, in 1000 iterations. For cross-validation using SVM, a nested three-fold cross-validation loop was used to choose from 0.1, 0.2, 0.5, 1, 2, 5, 10, 20 and 50 the best value for the kernel parameter *cost*, and the maximum number of microRNA variables was 15, with variable-filtering based on differential expression using limma’s moderated t-statistics. For cross-validation using TSP, the microRNA pair with the best TSP score constituted the variables.

### MicroRNA Quantification by Reverse Transcription-PCR (RT-PCR)

TaqMan® microRNA assays [43] from Applied Biosystems® (Foster City, CA) were used to quantify microRNAs *let-7e*, *miR-22*, *miR-30a-5p*, *miR-185*, *miR-210*, and *miR-423-5p* (assay identification numbers of 2406, 398, 417, 2271, 512, and 2340, respectively). Briefly, TaqMan® microRNA reverse transcription kit (Applied Biosystems®) was used to reverse transcribe 15 ng of RNA using a microRNA-specific oligonucleotide. PCR with real-time fluorometry was performed on RT reactions in triplicate in a 7900HT thermocycler. SDS software (version 2.4; Applied Biosystems®) was used to identify quantification cycle (C_q_) values and the mean C_q_ values for the triplicate PCRs were used for analysis. MicroRNA quantification of all RNA samples were performed in the same experiment. Negative control reactions, without any RNA, had undetectable C_q_ values.

### Other

All analyses were done in the Mac OS X 10.6 operating system. Annotated codes used in R for data processing, and differential expression and classification analyses are provided in text S1. Graphical plots were generated using R or Prism® (GraphPad Software®, La Jolla, CA; version 5.0d). Unless otherwise specified or implicit, all statistical tests were two-tailed, assumed equal group variances, and had a threshold of 0.05 for P value to identify significance. Receiver-operator characteristic curves were generated and areas under curves (AUC) determined using Prism® or R. Comparison of curves was performed online using StAR [44]. Analysis of differential expression using the Wilcoxon rank sum (Mann Whitney) test, and of hierarchical clustering of samples using log_2_-transformed microarray signals for expressed microRNAs, with Pearson correlation coefficient for distance metric and average linkage for inter-cluster distance, and with leaf-ordering of either the sample tree or the gene tree optimized, were done in TM4 [45] MultiExperiment Viewer (version 4.6 or 4.8). Processed microRNA expression data from the studies of Keller, et al. [31] and Leidinger, et al. [34] were obtained from the Gene Expression Omnibus database with accession numbers GSE17681 and GSE24709, respectively, and used directly for differential expression analyses.

## Results

### Clinical Characteristics of Cases and Controls

Clinical and demographic features of the 22 cases and 23 controls are summarized and detailed in tables 1 and S1, respectively. All cases had lung adenocarcinoma with pathological stage varying from IA to IIIB and were treated with surgical resection. Two cases had a second cancer, one with a synchronous lung cancer and the other with small lymphocytic lymphoma. The 23 controls were chosen for clinical relevance. Ten (43%) underwent surgical resection for a suspicious lung nodule or mass that was later found to be benign on pathological evaluation. The remaining 13 controls were at high risk for developing lung cancer because of age (>50 years) and a cigarette smoking history of >20 pack-years. There were no significant differences between cases and controls for gender distribution, smoking status, or blood hemoglobin level, WBC count or platelet count (table 1). However, there was a difference in age, with cases an average of 10.7 years older than the controls (P<0.01). There was no significant Pearson correlation between age and blood hemoglobin level, WBC count or platelet count.

**Table 1:** Demographic and clinico-pathologic characteristics of the study population.

Variable		Cases	Controls	P value^a^
Number		22	23	
Mean Age (Range, SD^b^)		70.6 (50–85, 7.7)	59.9 (36–74, 9.1)	<0.01
Gender (% Male)		55	52	1.00
Race (% Caucasian)		100	100	1.00
Tobacco Use (% Positive)		91	83	0.67
Cancer Stage	IA	9 (41%)		
	IB	5 (23%)		
	IIB	2 (9%)		
	IIIA	3 (14%)		
	IIIB	3 (14%)		
Histology	Adenocarcinoma	22 (100%)		
	Granuloma		1 (4%)	
	Hamartoma		7 (30%)	
	Fibrosis		1 (4%)	
	Amyloid		1 (4%)	
	Normal (No nodule)		13 (57%)	
Blood parameters (mean, SD)	White Blood Cell (x1000/µl)	6.7 (1.8)	7.7 (2.7)	0.17
	Platelets (x1000/µl)	233.9 (81.1)	263.4 (68.8)	0.24
	Hemoglobin (g/dl)	13.5 (1.4)	13.5 (1.3)	0.93

a Fisher’s exact test for categorical variables; two-tailed t tests assuming equal group variances for continuous variables.

b Standard deviation.

### Quantification of MicroRNAs in RNA Isolated from Whole Blood

Whole blood from the 45 cases and controls was collected in PAXgene™ Blood tubes, and total RNA isolated using the PAXgene™ Blood miRNA kit. The widely used PAXgene™ system incorporates cell lysis, RNA stabilization, and treatment with deoxyribonuclease for reproducible RNA purification and quantification [46], although some studies indicate that other blood collection and RNA isolation methods perform better [47], [48], [49]. Cases and controls did not differ for the µg of RNA isolated from 2.5 ml of blood, with overall mean being 2.65 (range = 1.25–5.26, standard deviation [SD] = 0.95). The mean of ratio of absorbances of the RNA isolates at 260 nm and 280 nm was 2.48 (range = 2.19–3.04, SD = 0.21), and of that at 260 nm and 230 nm was 0.21 (range = 0.09–0.44, SD = 0.08). There was no significant difference between cases and controls for the three parameters. There was no significant Pearson correlation between RNA yield and age, or blood hemoglobin, WBC count or platelet count.

A two-color, oligonucleotide [35] microarray platform from Exiqon® was used to quantify levels of 1282 human microRNAs and 23 human non-microRNAs of <200 nucleotides in the RNA isolated from whole blood specimens. As per version 18 of the miRBase microRNA repository [50], 1921 mature human microRNAs have been identified as of November 2011. The 415 RNAs deemed as expressed in >25% of the 45 samples of the study were used to generate the final microRNA expression profiles analyzed here. The 415 expressed RNAs included 20 (87%) of the 23 non-microRNAs, and 395 (31%) of the 1282 microRNAs, including 75 (20%) of the 376 miRPlus™ (Exiqon®) proprietary microRNA sequences, that were quantifiable with the microarrays. Descriptive statistics for the microarray signal values for the 415 RNAs are provided in table S2. About 57% and 87% of them were considered expressed in all and >50% of the 45 samples, respectively, as per the aforementioned criterion. The microarray signal values for the RNAs varied by about 9 log_2_ units though the 25th and 75th percentiles were about 2^5.5^ and 2^7.5^, respectively. There was no difference between cases and controls for the microarray signal value distributions (figure S1).

### Validation of Microarray-based MicroRNA Quantifications Using RT-PCR

**Figure 1:**
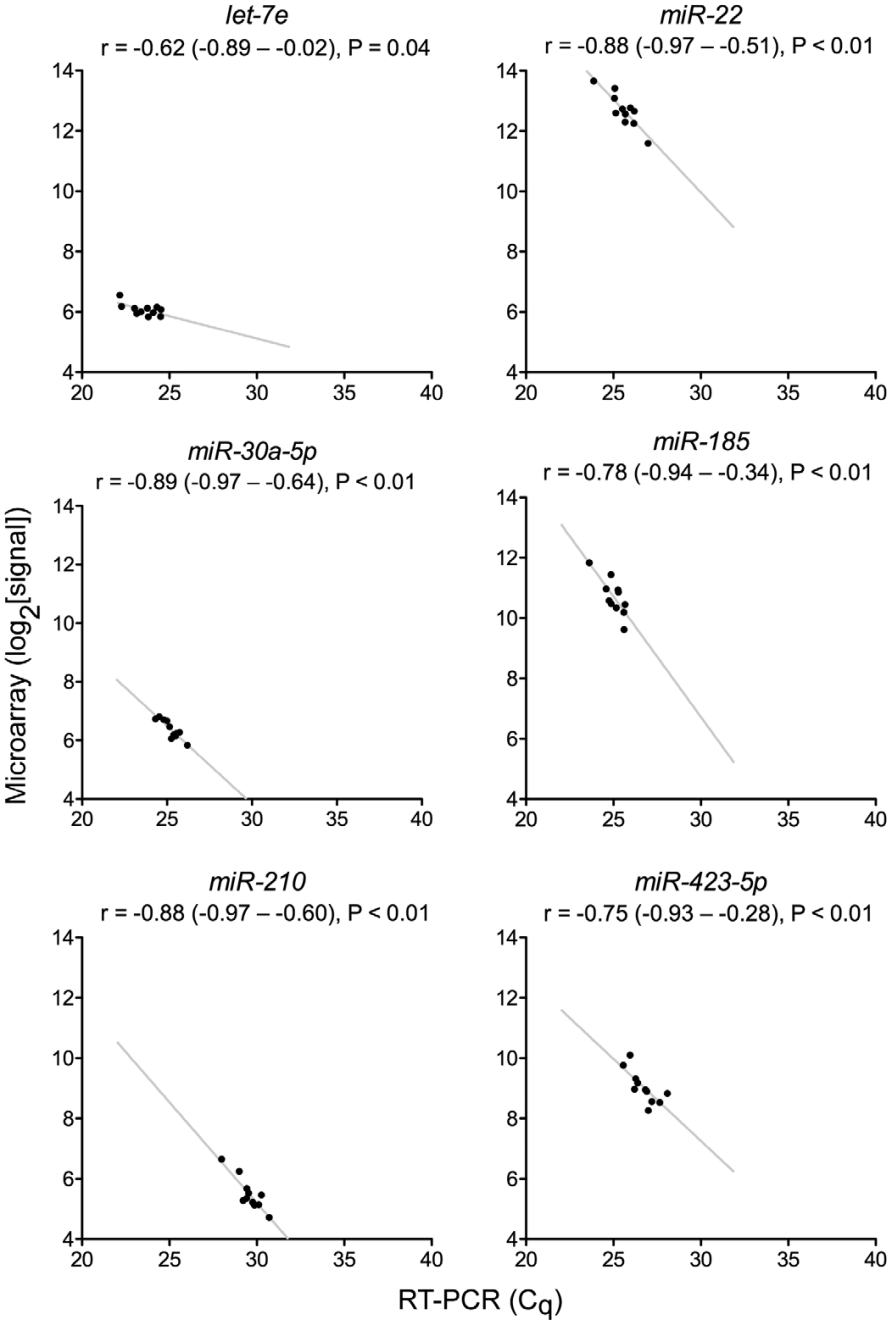
Correlation between microRNA quantification by reverse transcription-PCR (RT-PCR) and microarray. The scatter-plots show RT-PCR quantification cycle (C_q_) values and log_2_-transformed microarray signal values for microRNAs *let-7e*, *miR-22*, *miR-30a-5p*, *miR-185*, *miR-210*, and *miR-423-5p* (n = 11). Pearson correlation coefficients (r) and their 95% confidence intervals and associated P values, and best fitting (least squares) lines are also shown.

To check the accuracy of the microRNA expression data-set generated using microarrays, eight randomly selected microRNAs in RNA samples from 11 randomly selected subjects were quantified using RT-PCR-based TaqMan® microRNA assays [43]. Six of the eight microRNAs, *let-7e*, *miR-22*, *miR-30a-5p*, *miR-185*, *miR-210*, and *miR-423-5p*, were detectable in more than half of the samples, and demonstrated significantly good Pearson correlation (|r| >0.6) between log_2_-transformed microarray signal and RT-PCR C_q_ values, indicating validity of the microarray-based microRNA quantification (figure 1). An examination of the ranges of the quantifications showed that for five microRNAs the inter-sample difference was amplified 1.4–3.3x in the RT-PCR method compared to the microarray method; it was slightly diminished (0.9x) for *miR-185*. Such a generally wider signal distribution in the TaqMan® microRNA RT-PCR assay compared to the Exiqon® locked nucleic acid microarray assay has been reported previously [51].

### Changes in Whole Blood MicroRNA Levels in Patients with Lung Adenocarcinoma

**Figure 2:**
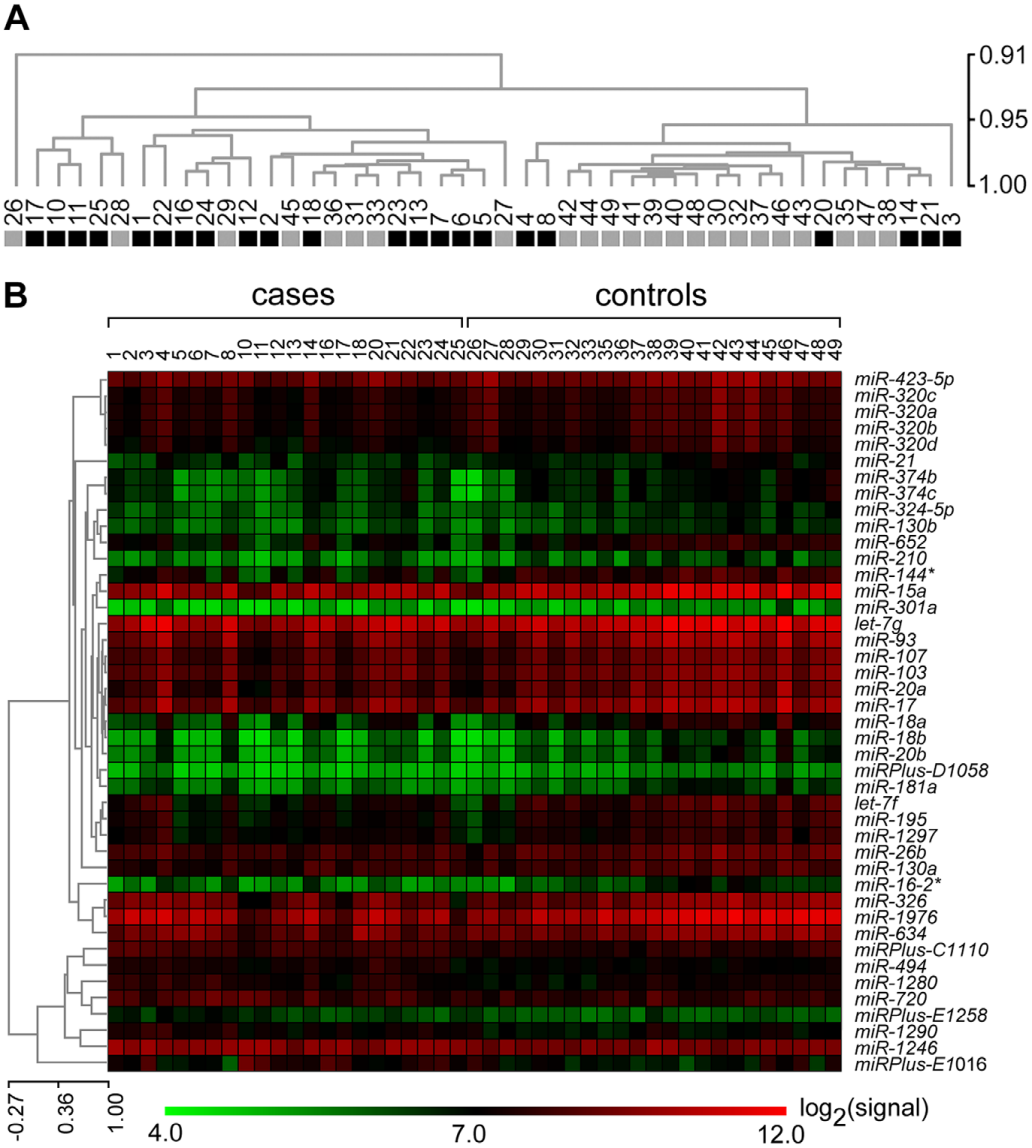
Whole blood microRNA expression in lung adenocarcinoma cases and controls. *A*. Unsupervised clustering of the 45 samples of this study by log_2_-transformed microarray signal values of all 395 expressed microRNAs. The numbers indicate identities of the 45 subjects, with cases (n = 22) and controls (n = 23) shown in black and grey, respectively. The sample tree with optimized leaf-ordering is drawn using Pearson correlation for distance metric and average linkage for cluster-to-cluster distance, and the scale for it represents node-heights. *B*. Supervised clustering of microRNAs by their log_2_-transformed microarray signal values. The heat-map, with the pseudo-color scale underneath, shows log_2_-transformed microarray signal values of the 43 microRNAs whose expression is altered >25% in either direction in the cases compared to the controls. The gene tree is drawn as in *A*.

Unsupervised hierarchical clustering using Pearson correlation measures of the quantification values for the set of 395 expressed microRNAs showed that there was a good clustering of the cases and controls, indicating presence of lung cancer-specific information in the microRNA expression profiles (figure 2A). This was supported by results of differential expression analyses. With the non-parametric Wilcoxon rank sum (Mann Whitney) test with P values adjusted for multiple testing by the Benjamini-Hochberg method for a false discovery rate of 5%, 122 (29%) of the 415 expressed RNAs, that included the 395 expressed microRNAs, were differentially expressed between cases and controls. Using empirical Bayes-moderated t-statistics calculated by the limma Bioconductor package [37], 104 (25%) of the 415 expressed RNAs were found to be differentially expressed with false discovery rate of <5% after Benjamini-Hochberg correction for multiple testing (table S2). Of the 104 RNAs, 102 (98%) were also identified as differentially expressed with the Wilcoxon test. The ratios of mean value for cases to that of controls (fold-change values) for the 104 differentially expressed RNAs that included 96 microRNAs ranged from 0.54 to 1.59. Among the 96 differentially expressed microRNAs, the expression of 47 was lower in cases compared to controls. Lists of 12 each of the differentially expressed RNAs with the most over- and under-expression values are shown in table 2. The relative expression of the 43 microRNAs whose expression was altered >25% in either direction in the cases compared to the controls is depicted as a heat map in figure 2B. Among the 23 controls, differential expression between those with pulmonary nodules and those without was seen for 198 (50%) of the 395 expressed microRNAs.

**Table 2:** Twelve differentially expressed small RNAs each that are most over- or under-expressed in cases compared to controls.

	Mean Expression (sd)^a^		
	Cases (n = 22)	Controls (n = 23)	Fold-Change
**Overexpressed RNAs**			
*miRPlus-E1016*	211.82 (129.47)	133.2 (53.13)	1.59
*SNORD2*	277.69 (156.92)	185.25 (36.39)	1.50
*SNORD44*	132.65 (65.03)	91.02 (17.84)	1.46
SNORD3@	602.26 (261.49)	415.53 (104.4)	1.45
*RNU5*	750.37 (310.9)	521.01 (111.97)	1.44
*RNU6–1*	552.41 (184.89)	390.61 (118.08)	1.41
*RNU6–1/RNU6–2* b	4037.13 (1552.97)	2878.75 (896.93)	1.40
*miRPlus-C1110*	312.42 (62.17)	224.99 (50.54)	1.39
*miRPlus-E1258*	86.75 (20.49)	64.07 (11.77)	1.35
*miR-720*	340.15 (119.55)	252.95 (74.57)	1.34
*miR-1290*	171.55 (50.99)	128.19 (32.76)	1.34
*SNORD13*	513.58 (204.28)	384.05 (88.93)	1.34
**Underexpressed RNAs**			
*miR-144*	143.57 (72.96)	267.88 (119.29)	0.54
*let-7f*	172.56 (96.41)	312.48 (150.58)	0.55
*miR-15a*	1067.1 (500.02)	1899.57 (859.99)	0.56
*miR-20a*	315.49 (215.14)	560.07 (295.46)	0.56
*miR-18a*	103.44 (62.86)	175.58 (100.54)	0.59
*miR-1976*	1141.76 (601.13)	1936.79 (853.76)	0.59
*miR-93*	585.97 (374.69)	980.62 (477.89)	0.60
*miR-20b*	50.43 (24.24)	83.2 (43.62)	0.61
*miR-320c*	207.32 (83.05)	339.03 (172.55)	0.61
*miR-17*	604.37 (343.17)	975.14 (443.34)	0.62
*miR-652*	126.41 (53.51)	202.94 (75.64)	0.62
*miR-18b*	44.08 (24.7)	69.15 (38.45)	0.64

a Microarray signal values are shown.

b Both *RNU6-1* and *RNU6-2* RNAs are detected by the same microarray probe.

### Ability of Whole Blood MicroRNA Expression Profiles to Distinguish Lung Adenocarcinoma Cases from Controls

Classification analyses with internal cross-validation were performed to determine if it was possible to distinguish cases from controls using whole blood microRNA expression profiles. Two different classification methods were employed: SVM with linear kernel, which has the advantage that there is only one adjustable kernel parameter (*cost*) to tune, and TSP, which is computationally simple, uses only two variables, is relatively unaffected by normalization methodology, and does not require differential expression of RNAs [42]. For SVM, variable filtering was done to use the 15 most differentially expressed microRNAs determined using limma’s moderated t-statistics, and an internal three-fold cross-validation was first performed to select the optimal value for *cost* to avoid biasing classification by adjusting this parameter on the test set [52]. In LOOCV, a classifier was generated using a training set of 44 samples and tested on the one remaining test sample, for a total of 45 possibly different classifiers and 45 predictions. In MCCV, the training and test sets had 36 and 9 samples, respectively, and the sets were randomly generated 1000 times, for a total of 1000 possibly different classifiers and 9000 predictions.

Using TSP, the prediction accuracy, sensitivity, and specificity determined in LOOCV were 96%, 91%, and 100%, respectively. MicroRNAs *miR-630* and *miR-1284* formed the best top-scoring pair and thus the classifier in all 45 iterations of LOOCV. A scatter-plot of the microarray signal values for the two microRNAs, of which only *miR-1284* is differentially expressed (table S2), shows the clear separation of cases and controls based on the ratios of these two microRNAs (figure S2). In MCCV, the means (and ranges and SDs) of prediction accuracy, sensitivity, specificity, were 92% (22–100, 13), 86% (0–100, 19), and 97% (0–100, 13), respectively. Thirty-five different microRNAs constituted the two-microRNA classifiers obtained in the 1000 iterations. MicroRNAs *miR-630* and *miR-1284*, also identified in the LOOCV analysis, were present in 947 and 918 of the classifiers, respectively, while the next most common microRNA was present in only 23. As expected, changing the sizes of training and test sets affected classifier performance (e.g., the mean accuracy increased from 75% at a training set-size of 12 to 95% at 42; figure S3).

**Figure 3:**
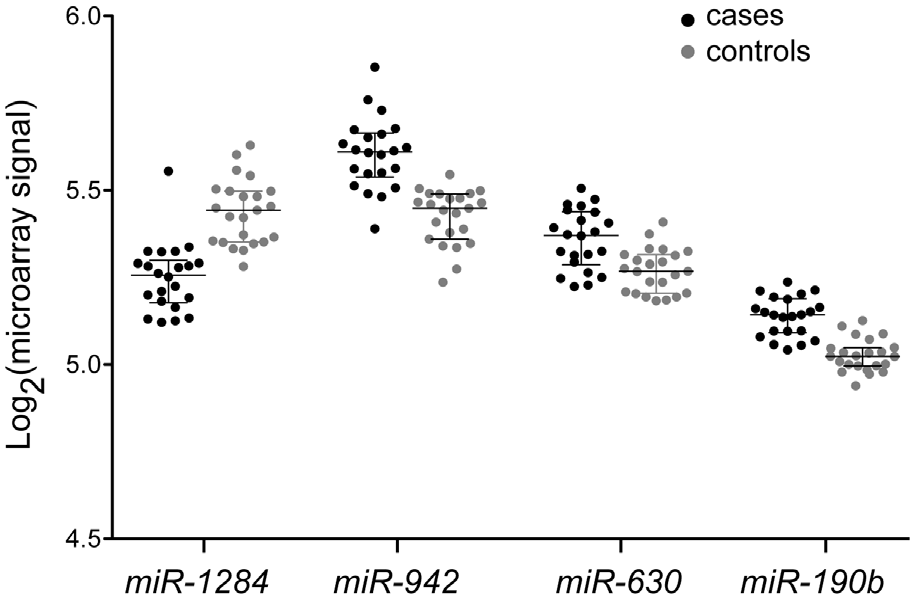
Expression of miR-1284, miR-942, miR-630, and miR-190b. Dot-plots with medians and inter-quartile ranges of log_2_-transformed microarray signal values for the 22 cases (*black*) and 23 controls (*grey*) are shown for the four microRNAs that are present in a majority of the classifiers generated in internal cross-validation analyses using the linear support vector machines and top-scoring pairs classification methods.

Using SVM, the prediction accuracy, sensitivity, and specificity values determined in LOOCV were all 91%. Twenty-four microRNAs were present in one or all of the 45 15-microRNA classifiers, eight (including *miR-1284*) of which were present in all. In MCCV, the means (with range and SD) of prediction accuracy, sensitivity, and specificity were 88% (44–100, 11), 88% (25–100, 17), and 89% (0–100, 16), respectively. Eighty-seven different microRNAs constituted the 15-microRNA classifiers obtained in the 1000 iterations. MicroRNAs *miR-190b*, *miR-942* and *miR-1284* were present in all of them. Changing the sizes of training and test sets affected classifier performance, though not as much as seen for TSP. For instance, increasing the training set size from 18 to 42 resulted in only a modest increase in mean accuracy, from 82% to 87% (figure S3). Overall, the two classification methods, SVM and TSP, identified four microRNAs (*miR-190b*, *miR-630, miR-942*, and *miR-1284*) that were present in a majority of the classifiers that were generated in the cross-validation analyses. The expression of these four microRNAs among the cases and controls is shown in figure 3.

### Effect of Age on MicroRNA Expression Profiles

Because of the significant difference in age between cases and controls (table 1), its effect on microRNA expression profile and its diagnostic utility was examined. The median age of the study population was used to separate it into cohorts of 22 young (age <68 years; 4 cancer cases and 18 controls) and 23 old (age ≥68 years; 18 cancer cases and 5 controls) subjects. Using limma’s t-statistics as described above, 65 (16%) of the 395 expressed microRNAs were identified as differentially expressed between the young and the old. Fifty-one (78%) of the 65 are among the 96 microRNAs differentially expressed between the lung cancer cases and controls, suggesting that age may have had a significant effect on the identification of microRNA expression differences between the cancer cases and controls.

**Figure 4:**
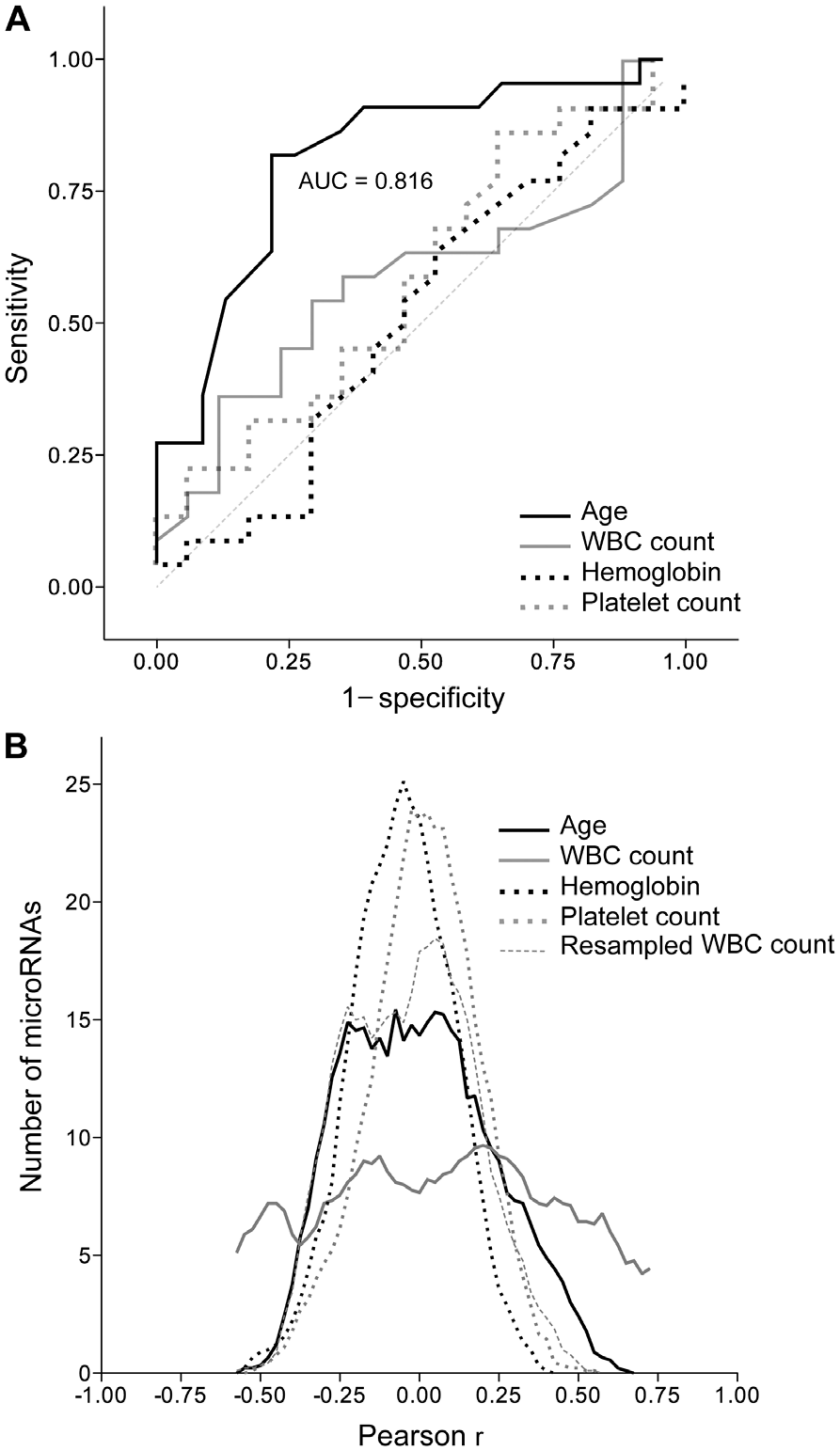
Association with lung adenocarcinoma of age, and blood hemoglobin level, and white blood cell (WBC) and platelet counts. *A*. Receiver operating characteristic curves, the areas under curve (*AUC*) for age, and the line of identity, *x* = *y*, with an AUC of 0.5, are shown. *B*. Correlation with microRNA expression. Values for the clinical variables were correlated with microarray signal values for the 395 expressed microRNAs (n = 45 for age; n = 39 for others). The curves depict frequency histograms of Pearson correlation coefficients (*r*) with a bin of 0.025. Curves were smoothened using four neighbors for averaging and a zero order polynomial. Correlations are also shown for the random variable *resampled WBC count* for which values were generated by resampling the WBC count data.

In Pearson correlation analyses of age and microarray signal values, though a significant correlation (|r|>0.4) between microarray signal values and age was seen for only 22 (6%) of the 395 expressed microRNAs, 20 (91%) of the 22 were differentially expressed between cancer cases and controls, and 12 (55%) of the 22 were among the 24 microRNAs present in one or all of the 45 15-microRNA classifiers obtained in LOOCV with the SVM method. In contrast, expression of 132 (33%) of the 395 expressed microRNAs, with 35 (27%) of the 132 among the 96 microRNAs differentially expressed between cancer cases and controls, correlated with the WBC count with |r|>0.4. For blood hemoglobin and platelet count, and for age if its values were resampled to simulate a random value distribution, |r|>0.4 was seen for 1.5%, 3% and 1.3% of the 395 expressed microRNAs, respectively (figure 4B).

To evaluate the effect of age further, receiver-operating characteristics analysis was used to determine if age could distinguish between cases and controls. Unlike blood parameter values for which AUCs were not significantly higher than 0.5, the AUC for age was 0.82 (figure 4A). This suggests that age has the potential to distinguish between cancer cases and controls. However, the AUC of 0.82 was significantly less (P<0.05 in the DeLong AUC comparison test [53]) than the AUC values of 0.95 and 1 seen respectively for ratios of microarray signal values for *miR-630* and *miR-1284*, the best top-scoring pair identified by the TSP method in the set of all 45 samples, and the probability values for being a case determined using a linear kernel SVM identified for the 45 samples (figure S4). Whether consideration of age along with microRNA expression data would improve classification was examined by receiver-operating characteristics analysis of the probability values obtained in LOOCV using the SVM method. The AUC without age being considered was 0.939 and it decreased slightly to 0.937 when age was included as a variable along with microRNA expression. Further, the prediction accuracy, sensitivity, and specificity, also declined slightly, by 2.2, 0, and 4.3 percentage units, respectively. This analysis, however, does not suggest that the diagnostic power in the microRNA expression profiles was uninfluenced by age because microRNA expressions were themselves affected by age.

Binary classification analysis with the TSP method in LOOCV showed that the microRNA expression profiles could be used to classify subjects into young (<68 years) or old with accuracy, sensitivity and specificity of 73%, 70% and 77%, respectively. With the SVM method, the values were 67%, 70% and 64%, respectively. As detailed earlier, prediction accuracy, sensitivity and specificity were all >90% for classification of subjects into cancer cases and controls. This suggests that the microRNA expression profiles, though likely influenced by age, had information content that could be used to separate cases and controls by their lung cancer status.

## Discussion

Changes in whole blood microRNA expression profiles because of diseases have been noted for both non-malignant conditions, such as myocardial infarction [28] and sarcoidosis [29], and cancers of tissues such as breast [24] and ovary [25]. This study sought to examine if such changes also occur in lung cancer. As referenced earlier, at least 14 studies have documented microRNA alterations in serum or plasma in lung cancer. The biological basis of such alterations remains unclear, and it is possible that it lies to at least some degree in the body’s systemic response and/or genetic susceptibility to cancer. If so, it might be manifested in changes in whole blood microRNA expression patterns. Compared to serum or plasma, whole blood is easier to collect and has 200–1000× more RNA content, which facilitates reliable and accurate global microRNA expression measurements using less clinical material. It should be noted that mature red blood cells (RBCs), whose cell concentration in blood is about 500× higher than that of WBCs and whose cellular mass per volume of blood is about 200× higher than that of platelets, bear a majority of whole blood microRNAs. MicroRNA concentration in mature RBCs is estimated to be similar to that in nucleated cells [54], and some microRNAs, such as *miR-16* and *miR-451* are present at more than a million-fold higher level in RBCs than plasma [55].

In this study, whole blood microRNA expression in lung cancer cases was compared to that in controls who did not have the disease but were clinically relevant because they had radiographically detected pulmonary nodules or were at high risk of developing lung cancer because of a significant smoking history (tables 1 and S1). Such types of subjects are commonly encountered in routine clinical practice and lung cancer screening programs. All the cases of this study had lung cancer of adenocarcinoma histology at pathologic stage IA-IIIB, and were similar to controls for history of smoking, gender, ethnicity, and blood hemoglobin levels, WBC and platelet counts (tables 1 and S1). Cigarette smoking is known to alter expression of circulating microRNAs [56], and changes in blood cell counts reflecting anemia, leukocytosis and thrombocytosis are frequently seen in lung cancer (e.g., [57], [58], [59]).

Significant differences in expression of 96 microRNAs were observed between the lung cancer cases and controls (figure 2B, and tables 2 and S2). These microRNAs included *miR-21* and *miR-210*, but not *miR-30a*, *miR-31*, *miR-126*, *miR-145*, or *miR-182*, all of which have been shown in multiple studies as differentially expressed between normal and cancerous lung tissues [60]. This discrepancy between microRNA expression changes in cancer tissues and in the circulating blood in lung cancer has been noted before [9], [61], and suggests that many of the differentially expressed microRNAs seen in this study do not originate from lung tissue. The changes in their levels likely reflect a systemic response or susceptibility to cancer. The 96 differentially expressed microRNAs of this study included microRNAs such as *miR-17* [9] and *miR-574-5p* [62], but not *miR-27b* [8] or *miR-155* [11], serum or plasma levels for all of which have been associated with presence of lung cancer. This observation can be expected from the difference in the types of cells that contribute to microRNA expression in whole blood and in extracellular circulation.

In unsupervised clustering analysis of the whole blood microRNA expression profiles, the cases and controls in this study segregated to a good degree (figure 2A). The biomarker potential of microRNA expressions to diagnose lung cancer was examined with internal cross-validations in classification analyses using two different methods (SVM and TSP), which yielded accuracy, sensitivity and specificity values ranging from 86% to 100%. Age was identified as a confounder for these results. The controls in this study were significantly younger than the cases (tables 1 and S1). The young and old subjects differed for the expression of 65 microRNAs, 78% of which were also identified as differentially expressed between cancer cases and controls. Of the 22 microRNAs whose expression had good correlation with age (figure 4B), 91% were differentially expressed between cancer cases and controls. However, in receiver-operating characteristics analyses, microRNA expression performed better at discerning cancer than age, with AUC values of 0.94 and 0.82 (figure 4A), respectively, and microRNA expression could classify lung cancer better than age in LOOCV analyses, with accuracy values of 91% and 67%, respectively. It thus appears that in spite of the effect of age, whole blood microRNA expression could be used to distinguish the lung cancer cases from the controls.

Four other studies have shown the association of changes in whole blood microRNA expression with lung cancer. However, there is minimal overlap between the significant microRNAs identified in these studies. For example, *let-7a* expression, identified as reduced in whole blood of lung cancer cases in the study of Jeong, et al. [33] was not significantly different between cases and controls in the current study and two other studies [32], [34]. Similarly, only eight and 10 of the differentially expressed microRNAs of the current study are also differentially expressed as per the studies of, respectively, Leidinger, et al. [34] and Keller, et al. [31]. MicroRNAs *miR-190b*, *miR-630, miR-942*, and *miR-1284*, the most frequent constituents of the classifiers generated in the current study, are not differentially expressed between cases and controls in the data-sets of either Keller, et al. or Leidinger, et al. as per the limma-based test used in the current study. Neither has been any of these microRNAs reported as differentially expressed between lung cancer cases and controls in a recent transcriptome sequencing study of whole blood microRNAs [32]. This low discordance between the findings of this study and the others could be a result of the different microRNA quantification platforms used in the studies, or could be because clinical and demographic profiles of the case and control cohorts vary significantly among these studies. For instance, the controls in the study of Keller, et al. are significantly younger than the cases whereas cases and controls are of similar age in the study of Jeong, et al. Similarly, the controls used in the Leidinger study were selected from a cohort of chronic obstructuve pulmonary disease patients while the controls in the study of Keller, et al. were all healthy.

Many of the controls in the current study did not undergo radiological investigations like computerized tomography whereas all the cases did. Radiation exposure, even at low dosage, has been shown to significantly affect levels of microRNAs in blood [63], [64], [65]. It is therefore possible that some of the changes in microRNA expression noted here are actually consequent to radiation exposure. Similar differences between the cases and controls for other environmental factors such as use of medications, many of which have been shown to influence blood microRNAs [66], [67], may also underlie the observations of this study. Blood microRNA expression profiles appear to reflect the physiological state of the body as well, as suggested by studies that have examined their correlations with age [68], blood pressure [66], diurnal state [69], gender [56], mental anxiety [70], physical stress [71], etc.

It is clear that one has to judge with good temperance the association of blood microRNAs with lung adenocarcinoma that is noted in this investigation, which is beset with small sample-size, significant age difference between cases and controls, and use of two types of controls. Additional studies with large sample sizes, and case and control cohorts matched for important variables such as age, gender, smoking status, and blood cell counts are required to confirm the association of whole blood microRNA changes with lung cancer. Identification of specific microRNA biomarkers for clinical utility will require the use of an appropriate and precisely defined control population. Comparison of microRNA expression before and after tumor resection may also be useful in identifying if these biomarkers can detect the presence of lung cancer or predict individual susceptibility.

## Supporting Information

Figure S1 **Scatter-plot of mean microarray signal values of expressed RNAs in the two cohorts.** Means for each of the 407 probes for which the target RNAs are considered expressed for the 22 cases are plotted against the means for the 23 controls (*black dots*). Some probes recognize multiple species of RNAs. Error lines indicating the standard deviations for the case and control cohorts are shown in red and green, respectively. The grey line represents *x = y*. Axes are on a log_2_ scale.
(TIF)

Figure S2 **Expression of miR-630 and miR-1284.** Microarray signal values for *miR-630* and *miR-1284* that constitute the best top-scoring pair (TSP) in TSP analysis of microRNA expression profiles of the 22 cases (*black*) and 23 controls (*grey*) are plotted.
(TIF)

Figure S3 **Effect of training-set size on performance of classifiers in Monte Carlo cross-validation analyses.** Mean and 95% confidence interval values for accuracy, sensitivity, specificity, and positive and negative predictive values of varying training-set sizes in Monte Carlo cross-validation analyses using the top-scoring pairs (*TSP*) or support vector machines (*SVM*, linear kernel) classifier methods are shown along the left *Y* axis. The total number of microRNAs constituting the 1000 classifiers generated for each training-set size is shown along the right *Y* axis. Analyses were performed as described in the *Material and methods* section for the particular case of a training-set size of 36.
(TIF)

Figure S4 **Receiver operating characteristic curves for top-scoring pairs (TSP) and support vector machines (SVM) classifier methods.** On left, the curve shows the association with the presence of lung adenocarcinoma of the ratio of microarray signals for *miR-630* and *miR-1284* that constitute the best pair of expressed microRNAs identified by the TSP method in the 45 samples of the study. On right, the variable is the probability for membership in the class of lung adenocarcinoma cases calculated from the best linear kernel SVM determined using all 45 samples of the study. Areas under curve (*AUC*) are also shown.
(TIF)

Table S1 Case-specific demographic and clinico-pathologic details.
(PDF)

Table S2 Descriptive statistics of microarray signal values for all expressed microRNAs and non-microRNA small RNAs.
(PDF)

Text S1 R codes for processing of microarray data, differential gene expression analysis using moderated t-statistics in limma Bioconductor package, leave-one-out cross-validation analyses with top-scoring pairs (TSP) and linear support vector machines (SVM) classification methods, and Monte Carlo cross-validation analyses with TSP and SVM, are shown with annotation and information about the computing platform and R packages.
(PDF)
