# Supplementary figures and images for: MicroRNA Expression Profiles of Whole Blood in Lung Adenocarcinoma
Source: PLoS One. 2012 Sep 28;7(9):e46045. doi: 10.1371/journal.pone.0046045 (PMC3460960; doi:10.1371/journal.pone.0046045)

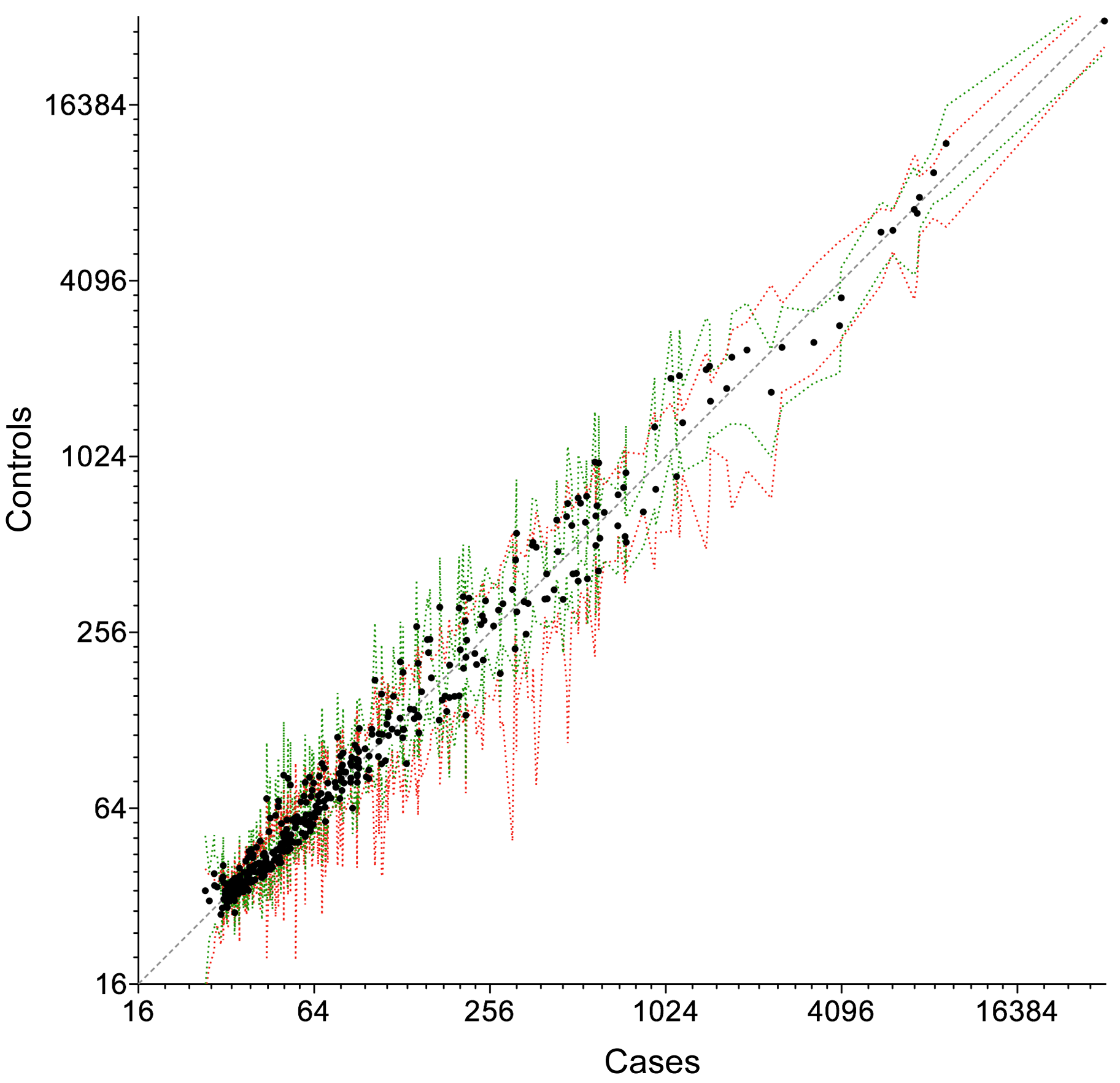

Supplement: Figure S1 — Scatter-plot of mean microarray signal values of expressed RNAs in the two cohorts. Means for each of the 407 probes for which the target RNAs are considered expressed for the 22 cases are plotted against the means for the 23 controls (black dots). Some probes recognize multiple species of RNAs. Error lines indicating the standard deviations for the case and control cohorts are shown in red and green, respectively. The grey line represents x = y. Axes are on a log2 scale. (TIF) [file pone.0046045.s001.tif]

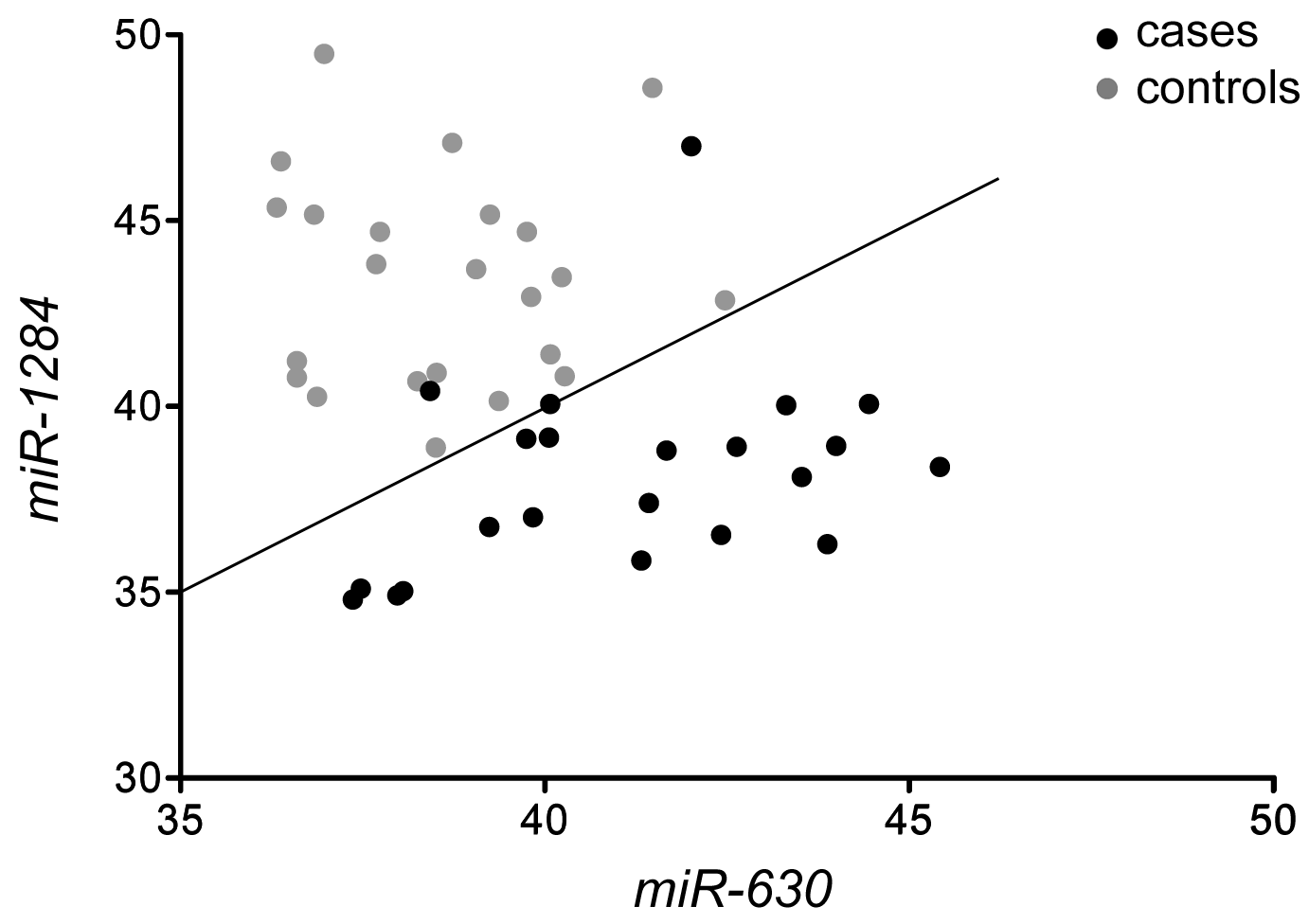

Supplement: Figure S2 — Expression of miR-630 and miR-1284. Microarray signal values for miR-630 and miR-1284 that constitute the best top-scoring pair (TSP) in TSP analysis of microRNA expression profiles of the 22 cases (black) and 23 controls (grey) are plotted. (TIF) [file pone.0046045.s002.tif]

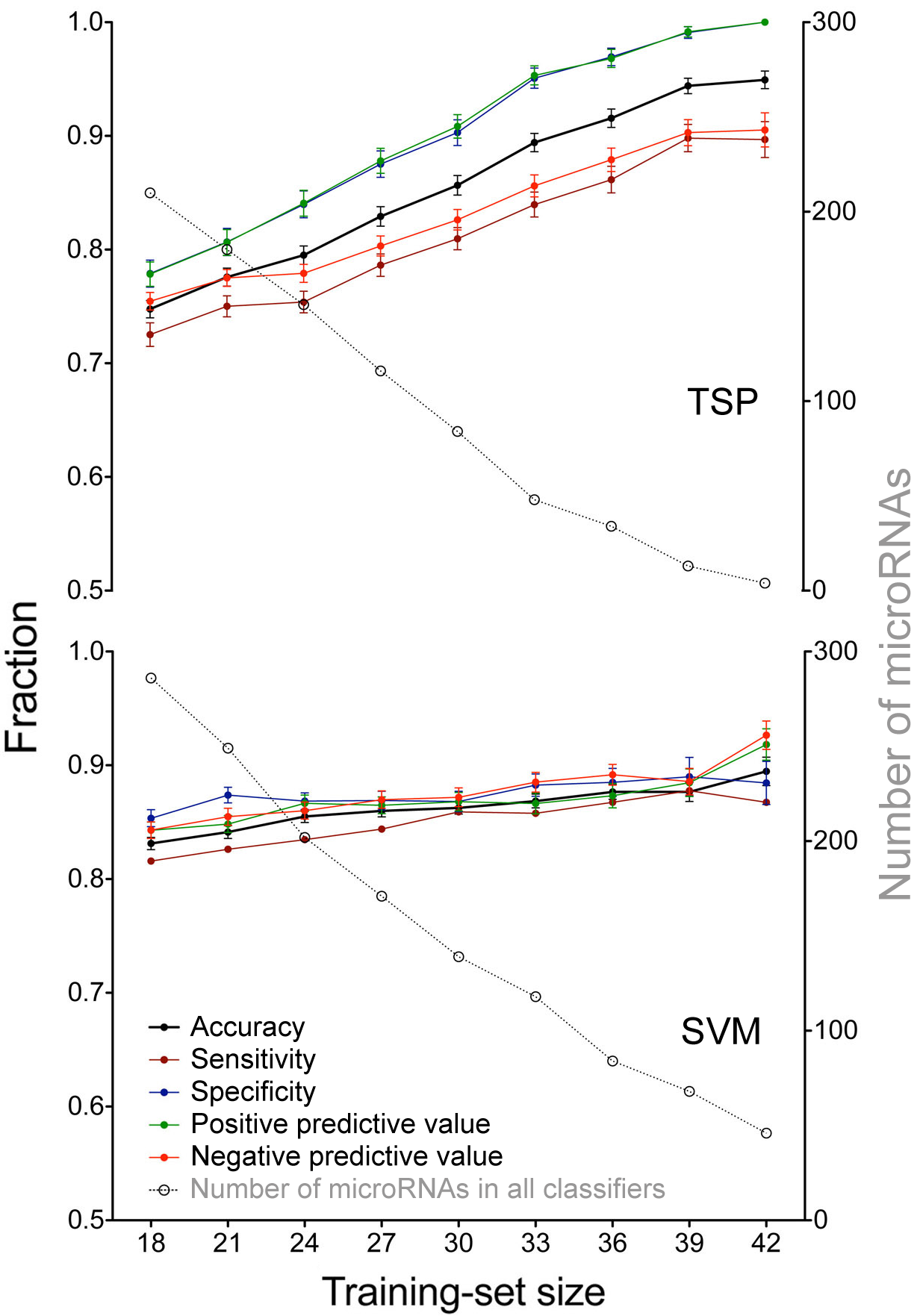

Supplement: Figure S3 — Effect of training-set size on performance of classifiers in Monte Carlo cross-validation analyses. Mean and 95% confidence interval values for accuracy, sensitivity, specificity, and positive and negative predictive values of varying training-set sizes in Monte Carlo cross-validation analyses using the top-scoring pairs (TSP) or support vector machines (SVM, linear kernel) classifier methods are shown along the left Y axis. The total number of microRNAs constituting the 1000 classifiers generated for each training-set size is shown along the right Y axis. Analyses were performed as described in the Material and methods section for the particular case of a training-set size of 36. (TIF) [file pone.0046045.s003.tif]

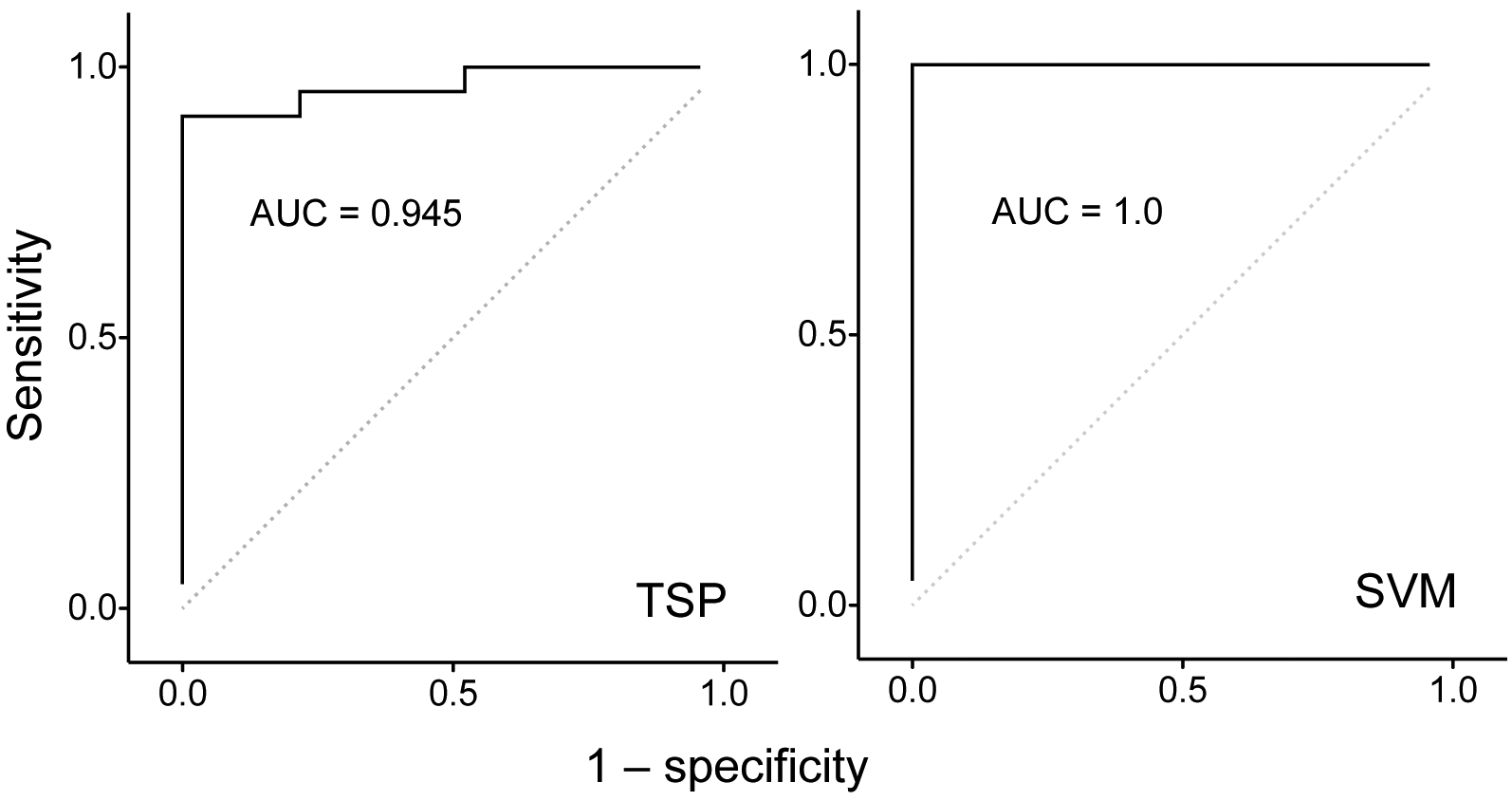

Supplement: Figure S4 — Receiver operating characteristic curves for top-scoring pairs (TSP) and support vector machines (SVM) classifier methods. On left, the curve shows the association with the presence of lung adenocarcinoma of the ratio of microarray signals for miR-630 and miR-1284 that constitute the best pair of expressed microRNAs identified by the TSP method in the 45 samples of the study. On right, the variable is the probability for membership in the class of lung adenocarcinoma cases calculated from the best linear kernel SVM determined using all 45 samples of the study. Areas under curve (AUC) are also shown. (TIF) [file pone.0046045.s004.tif]
