# Supplementary material for: MicroRNA Expression Profiles of Whole Blood in Lung Adenocarcinoma
Source: PLoS One. 2012 Sep 28;7(9):e46045. doi: 10.1371/journal.pone.0046045 (PMC3460960; doi:10.1371/journal.pone.0046045)
Supplement: Table S1 — Case-specific demographic and clinico-pathologic details. (PDF) [file pone.0046045.s005.pdf]

Table S1

Case-specific demographic and clinico-pathologic details[a]

ID	Histology of lung tumor	Age (y)	Sex	History of smoking	Current smoker	Duration of last smoking cessation	Pathologic stage of lung cancer	Year of surgery	Days before surgery that blood was collection for blood tests, or year of blood collection	WBC (x1000/µl)	Hgb (g/dl)	Platelets (x1000/µl)	Note
1	AC	76	F	Yes	Yes		T1N2M0	2007	0	5.7	13.5	299	
2	AC	73	M	Yes	No	22 y	T2N0M0	2007	24	7	15.8	177	
3	AC	69	M	Yes	No	3 y	T1N0M0	2007	26	9.3	14.9	376	
4	AC	68	M	Yes	No	16 y	T1N2M0	2007	0	8.6	13.6	156	Post-operative blood collection
5	AC	58	M	Yes	No	1 y	T2N2M0	2007	7	3.4	12	55	
6	AC	72	F	Yes	No	1 y	T2N1M0	2007	24	5.6	14	328	Has synchronous stage IA lung AC
7	AC	83	M	Yes	No	31 y	T1N0M0	2008	26	4.7	13.2	221	
8	AC	77	M	Yes	No	29 y	T1N0M0	2008	12	6	13.8	214	
10	AC	72	F	No	No		T1N0M0	2009	22	8.8	13.7	266	
11	AC	68	F	No	No		T2N0M0	2009	31	5.2	14.3	238	
12	AC	75	F	Yes	No	1 y		2009	5	8	13.1	277	Has multifocal disease
13	AC	85	M	Yes	No	41 y		2009	0	4.5	11	97	Has small lymphocytic leukemia
14	AC	72	M	Yes	No	11 y	T2N1M0	2009	13	8.9	13.4	206	
16	AC	64	F	Yes	Yes		T1N0M0	2009	20	8.9	13.2	380	
17	AC	69	F	Yes	No	45 y	T1N1M0	2009	13	7.3	14	275	
18	AC	50	F	Yes	Yes		T1N0M0	2009	20	5.3	13.3	239	
20	AC	72	M	Yes	No	25 y	T2N0M0	2009	13	5.9	14.3	125	
21	AC	68	F	Yes	No	38 y	T1N0M0	2009	13	6.2	13.9	250	
22	AC	63	M	Yes	No	20 y	T1N0M0	2009	20	6.6	10.1	248	
23	AC	78	M	Yes	No	41 y	T4N0M0	2009	2	8.3	12.7	203	
24	AC	73	M	Yes	No	33 y	T4N2M0	2009	18	8.9	16.6	218	
25	AC	68	F	Yes	No	14 y	T1N0M0	2010	371	4.9	12.6	297	
26	Granuloma	61	F	No	No			2007	12	5.8	14.4	249	
27	Granuloma	45	M	Yes	No	3 w		2009	16	5.1	15.6	269	
28	Normal	74	F	Yes	No	2 y		2009	26	7.4	11.4	306	
29	Hamartoma	36	F	Yes	No	20 y		2009	6	7.1	12.8	223	
30	Amyloid	69	M	Yes	No	22 y		2009	1	8.3	13.2	216	
31	Granuloma	63	F	No	No			2009	15	6.8	14.6	308	
32	Granuloma	69	M	Yes	No	5 y		2009	7	8.6	15.3	213	

33	Granuloma	72	M	Yes	No	30 y	2009	13	4.7	13.4	234	
35	Granuloma	51	F	Yes	No	1 m	2010	17	8.3	13.1	189	
36	Granuloma	64	F	No	No		2010	24	5.8	13.8	210	
37		58	F	Yes	No	3 m		2009	10.6	13.2	354	No lung nodule; blood collected >90 d before surgery
38		59	F	No	No			2007	6.1	13.6	277	Lung nodule radiologically-stable for 2 y; blood collected >90 d before surgery
39		55	F	Yes	No	20 y						No lung nodule
40		65	M	Yes	No	10 y						No lung nodule
41		54	M	Yes	No	3 m		2005	8.2	16	382	No lung nodule; blood collected >90 d before surgery
42		60	M	Yes	No	20 y		2008	7.5	12.6	354	No lung nodule; blood collected >90 d before surgery
43		54	M	Yes	Yes							No lung nodule
44		61	F	Yes	No	42 y						No lung nodule
45		74	M	Yes	No	15 y						No lung nodule
46		64	M	Yes	No	8 y		2007	16.4	11.6	351	No lung nodule; blood collected >90 d before surgery
47		55	M	Yes	Yes							No lung nodule
48		62	M	Yes	No	20 y		2010	7.3	13.9	188	No lung nodule; blood collected >90 d before surgery
49		53	M	Yes	Yes			2010	7.4	11.7	154	No lung nodule

[a]Individuals with IDs <26 belong to the case cohort, and others belong to the control cohort. Cells for unnavailable data or unapplicable variables are left blank. AC, adenocarcinoma; d, day; F, female; Hgb, hemoglobin; m, month; M, male; WBC, white blood cell; y, year. All individuals are of white race.
