# Supplementary material for: MicroRNA Expression Profiles of Whole Blood in Lung Adenocarcinoma
Source: PLoS One. 2012 Sep 28;7(9):e46045. doi: 10.1371/journal.pone.0046045 (PMC3460960; doi:10.1371/journal.pone.0046045)
Supplement: Table S2 — Descriptive statistics of microarray signal values for all expressed microRNAs and non-microRNA small RNAs. (PDF) [file pone.0046045.s006.pdf]

Table S2

Descriptive statistics of microarray signal values for all expressed microRNAs and non-microRNA small RNAs[a]

RNA	*Cases (n = 22)*		*Controls (n = 23)*					
	Mean (range; standard deviation)	*Median*	*Mean (range; standard deviation)*	*Median*	*Fold-change*	*Moderated t-statistic*	*Moderated F-statistic*	*Adjusted P*
			**MicroRNAs**					
let-7a	1724.49 (720.25-4564.11; 1048.68)	1363.65	2244.31 (974.25-4202.21; 914.23)	1986.04	0.77	-1.79	3.19	0.17512
let-7b	306.04 (143.41-1147.12; 256.53)	205.05	359.4 (136.86-741.44; 162.58)	341.25	0.85	-0.84	0.71	0.51844
*let-7b**	50.55 (34.19-62.75; 8.19)	52.40	50.88 (30.08-64.85; 8.14)	51.94	0.99	-0.13	0.02	0.92430
let-7c	55.37 (34.91-194.17; 36.04)	44.73	56.66 (35.3-98.51; 13.93)	55.01	0.98	-0.16	0.03	0.91062
let-7d	469.02 (195.76-1419.2; 296.72)	369.01	638.75 (276.2-1129.54; 237.52)	658.44	0.73	-2.14	4.57	0.10641
*let-7d**	81.06 (54.15-137.47; 19.52)	78.20	78.06 (65.44-125; 12.75)	75.47	1.04	0.62	0.38	0.64058
let-7e	71.03 (55.54-123.53; 14.76)	68.04	69.3 (50.75-96.04; 10.64)	69.49	1.02	0.45	0.21	0.73257
let-7f	172.56 (68.18-475.14; 96.41)	154.40	312.48 (57.72-677.46; 150.58)	331.53	0.55	-3.72	13.83	0.00723
let-7g	1446.88 (584.58-3361.89; 703.15)	1237.61	2089.45 (912.3-3982.66; 848.14)	1888.30	0.69	-2.78	7.72	0.03706
let-7i	472.98 (158.83-1539.37; 365.86)	339.13	707.53 (267.4-1721.13; 402.96)	619.39	0.67	-2.06	4.23	0.11617
*let-7i**	29.19 (20.78-44.75; 6.56)	26.36	34.8 (22.98-55.91; 8.92)	35.30	0.84	-2.40	5.77	0.07058
miR-1	45.26 (31.12-72.03; 10.26)	42.65	39.56 (28.57-65.66; 8.67)	37.15	1.14	2.02	4.09	0.12296
miR-100	41.52 (23.06-61.61; 11.53)	39.72	41.56 (24.06-71.99; 11.69)	40.23	1.00	-0.01	<0.01	0.99121
miR-101	129.15 (50.65-276.71; 68.2)	103.96	186.76 (73.36-439.94; 81.46)	172.17	0.69	-2.58	6.68	0.05066
miR-103	434.09 (196.42-830.87; 181.77)	376.72	620.84 (189.64-990.97; 230.98)	614.98	0.70	-3.03	9.15	0.02518
*miR-103-2**	71.49 (50.07-121.92; 18.83)	65.50	71.29 (32.29-99.89; 16.46)	74.64	1.00	0.04	<0.01	0.97550
miR-106a	1409.83 (398.99-3814.5; 916.07)	1010.05	2034.3 (503.75-4879.82; 1027.56)	2016.76	0.69	-2.16	4.68	0.10175
miR-106b	938.82 (285.53-1904.85; 516.56)	662.68	1293.39 (407.56-3124.43; 614.46)	1171.02	0.73	-2.10	4.43	0.11003
*miR-106b**	210.96 (71.05-397.55; 84.88)	189.32	280.13 (75.52-494.16; 108.32)	297.92	0.75	-2.39	5.73	0.07083
miR-107	360 (140.73-709.75; 165.9)	311.01	521.81 (162.81-948.43; 206.41)	569.90	0.69	-2.91	8.47	0.03034
miR-10a	51.68 (35.72-69.6; 9.77)	51.83	48.94 (31.36-65.83; 8.43)	47.63	1.06	1.01	1.03	0.44729
miR-10b	39.39 (28.29-57.78; 7.6)	37.52	35.93 (25.43-52.68; 6.88)	36.63	1.10	1.60	2.58	0.21963
miR-1184	80.03 (34.02-208.98; 39.99)	69.51	89.94 (50.04-152.74; 29.16)	87.97	0.89	-0.96	0.92	0.46181
miR-1207-3p	64.67 (55.12-71; 4.55)	64.93	61.89 (51.82-88.36; 7.26)	61.47	1.04	1.53	2.35	0.24165
miR-122	51.93 (35.35-88.09; 12.53)	48.30	45.71 (32.23-80.32; 11.75)	42.40	1.14	1.73	2.99	0.18753
miR-1227	48.95 (43.74-53.4; 2.53)	48.47	46.95 (41.25-65.25; 4.53)	45.99	1.04	1.80	3.26	0.17253
*miR-1228**	33.2 (29.94-35.66; 1.54)	33.30	32.66 (28.71-36.62; 1.78)	32.28	1.02	1.01	1.03	0.44729
miR-1236	57.65 (47.23-79.55; 6.89)	56.65	58.22 (48.82-68.43; 5.63)	57.54	0.99	-0.30	0.09	0.83345
miR-124	46.04 (30.8-93.1; 13.67)	41.48	40.24 (27.93-78.03; 11.33)	38.00	1.14	1.56	2.44	0.23636
miR-1246	859.35 (470.08-1147.21; 183.81)	859.03	662.12 (422.13-1152.96; 160.66)	655.42	1.30	3.86	14.93	0.00553
miR-1247	32.82 (29.59-35.41; 1.57)	32.56	34.5 (30.21-40.38; 2.28)	34.47	0.95	-2.73	7.45	0.04119
miR-1248	34.28 (23.15-52.3; 7.52)	33.08	28.06 (21.25-33.27; 3.13)	27.89	1.22	3.65	13.32	0.00836

miR-1249	106.85 (85.52-118.36; 8)	108.53	108.69 (92.24-125.49; 9.19)	108.76	0.98	-0.72	0.51	0.58292
miR-1250	33.71 (29.89-36.43; 1.56)	34.10	33.58 (29.49-55.55; 5.05)	32.19	1.00	0.11	0.01	0.92789
miR-1255a	85.44 (72.95-97.34; 6.56)	86.18	78.73 (64.55-85.35; 5.72)	80.29	1.09	3.67	13.44	0.00820
miR-125a-5p	123.47 (91.7-176.05; 23.31)	121.00	116.31 (83.25-182.94; 25.32)	109.17	1.06	0.99	0.99	0.45169
miR-125b	185.92 (66.38-428.96; 100.03)	173.28	153.3 (68.9-343.94; 71.64)	148.68	1.21	1.27	1.61	0.33743
miR-126	357.93 (146.67-1040.09; 208.28)	278.30	509.01 (183.55-1017.08; 228.16)	456.72	0.70	-2.33	5.44	0.07800
miR-126*	38.98 (32.42-55.04; 4.46)	38.11	46.18 (36.71-67.77; 7.67)	44.04	0.84	-3.83	14.70	0.00564
miR-1261	78.76 (42.89-156.15; 32.08)	69.04	96.65 (63.19-165.61; 24.86)	93.10	0.81	-2.11	4.45	0.10958
miR-1264	89.96 (49.01-168.35; 24.02)	84.46	83.42 (50.77-133.44; 22.23)	84.61	1.08	0.96	0.91	0.46295
miR-1265	35.73 (30.42-41.04; 2.37)	35.22	34.97 (29.42-41.58; 3.05)	34.79	1.02	0.91	0.82	0.48782
miR-1268	38.81 (35.32-44.04; 2.13)	38.30	35.67 (32.94-41.73; 1.96)	35.44	1.09	4.93	24.29	0.00063
miR-1274a	50.42 (39.54-67.83; 7.6)	49.29	45.64 (37.35-62.69; 6.03)	43.89	1.10	2.35	5.51	0.07578
miR-1274b	34.48 (29.39-37.19; 2.18)	34.90	33.68 (29.92-35.75; 1.78)	34.35	1.02	1.30	1.70	0.32519
miR-1275	50.24 (38.34-70.91; 8.18)	49.05	47.53 (29.34-62.62; 9.26)	46.34	1.06	1.04	1.08	0.43720
miR-1279	32.42 (28.95-34.65; 1.45)	32.69	31.49 (29.81-33.72; 1.04)	31.16	1.03	2.18	4.77	0.09804
miR-128	67.69 (54.62-96.81; 11.13)	64.85	71.94 (47.98-87.48; 10.42)	71.40	0.94	-1.33	1.77	0.31338
miR-1280	201.19 (113.84-314.92; 60.28)	196.40	155.33 (97.27-220.03; 35.33)	156.33	1.30	3.15	9.94	0.02040
miR-1281	69.81 (60.53-79.15; 4.35)	70.09	64.46 (55.92-96.27; 10.13)	62.12	1.08	2.29	5.25	0.08416
miR-1284	38.12 (34.8-47; 2.71)	38.23	43.43 (38.9-49.49; 2.85)	43.48	0.88	-6.26	39.20	0.00002
miR-1285	54.93 (29.46-80.13; 13.14)	53.72	59.29 (33.28-90.15; 13.04)	59.25	0.93	-1.12	1.26	0.39965
miR-129-5p	283.68 (117.62-733.73; 156.99)	247.69	321.18 (180.28-513.17; 89.58)	326.41	0.88	-1.00	0.99	0.45160
*miR-129**	43.21 (31.21-64.25; 7.04)	41.33	44.95 (33.44-54.01; 5.75)	45.50	0.96	-0.91	0.83	0.48782
miR-1290	171.55 (107.34-298.72; 50.99)	158.79	128.19 (91.04-218.49; 32.76)	120.01	1.34	3.43	11.78	0.01280
miR-1297	157.84 (93.03-353.69; 57.39)	146.54	218.44 (68.98-417.83; 89.55)	195.78	0.72	-2.71	7.33	0.04245
miR-1299	61.68 (48.2-71.09; 5.43)	62.03	57.96 (43.19-68.21; 5.8)	57.13	1.06	2.22	4.92	0.09291
miR-1301	32.7 (28.99-36.5; 2.18)	32.61	34.4 (28.19-41.45; 2.75)	34.07	0.95	-2.23	4.97	0.09158
miR-1304	38.36 (32.93-45.32; 2.72)	38.11	38.26 (33.65-42.14; 2.17)	38.75	1.00	0.13	0.02	0.92430
miR-130a	247.32 (144.38-432; 88.18)	226.91	328.87 (169.99-518.97; 101.64)	293.32	0.75	-2.89	8.35	0.03129
miR-130b	61.75 (41.49-93.76; 16.88)	54.83	81.8 (38.13-131; 23.46)	82.87	0.75	-3.30	10.88	0.01639
*miR-130b**	32.86 (28.33-37.09; 1.9)	33.01	33.43 (26.87-36.97; 2.49)	34.42	0.98	-0.84	0.70	0.52195
miR-1321	33.09 (29.49-37.42; 2.52)	33.30	32.4 (26.67-35.39; 2.1)	32.65	1.02	0.97	0.94	0.46135
miR-1323	31.04 (23.76-42.66; 4.71)	31.03	29.13 (22.88-36.89; 4.09)	28.62	1.07	1.45	2.10	0.26887
miR-133a	34.19 (23.67-60.6; 8.68)	32.96	30.92 (21.77-51.07; 7.29)	29.17	1.11	1.38	1.89	0.29310
miR-133b	42.35 (29.44-70.8; 10.02)	40.33	37 (23.14-63.4; 10.43)	34.89	1.14	1.76	3.10	0.18095
miR-135a	36.79 (31.55-44.38; 3.36)	36.00	35.83 (29.88-41.18; 3)	35.10	1.03	1.00	1.01	0.45039
miR-138-1*	52.33 (40.24-61.55; 5.44)	51.70	47.9 (38.84-57.94; 4.95)	49.03	1.09	2.86	8.15	0.03217
miR-140-3p	1657.25 (716.94-2873.36; 662.44)	1501.73	1753.58 (692.32-2690.82; 452.46)	1699.03	0.95	-0.58	0.33	0.66343
miR-141	43.84 (30.81-60.92; 8.46)	43.05	39.29 (29.92-61.31; 7.72)	39.22	1.12	1.89	3.57	0.15210
miR-142-3p	242.76 (86.96-706.94; 133.48)	208.08	205.54 (116.65-405.93; 71.95)	186.34	1.18	1.18	1.39	0.37332
miR-142-5p	244.49 (83.09-460.39; 104.77)	258.56	281.32 (59.41-514.17; 95.49)	263.26	0.87	-1.24	1.54	0.34983
miR-143	59.73 (37.42-96.28; 13.55)	57.51	52.33 (29.78-75.36; 13.07)	52.84	1.14	1.88	3.52	0.15583
*miR-143**	33.55 (30.25-36.61; 1.91)	33.33	32.2 (27.6-37.03; 2.01)	32.26	1.04	2.19	4.81	0.09638
miR-144	145.27 (40.29-347.28; 84.76)	130.56	200.57 (57.36-377.16; 84.77)	181.98	0.72	-2.20	4.85	0.09511

miR-144*	143.57 (46.7-295.85; 72.96)	145.66	267.88 (47.73-517.8; 119.29)	265.76	0.54	-4.22	17.83	0.00259
miR-145	63.65 (41.61-95.91; 14.98)	58.81	56.33 (37.45-87.42; 12.88)	56.16	1.13	1.77	3.14	0.17999
miR-1469	58.97 (44.19-81.82; 10.26)	58.16	58.66 (48.2-79.2; 7.45)	57.50	1.01	0.12	0.01	0.92628
miR-146a	38.29 (28.6-51.29; 7.18)	36.06	45.58 (30.76-61.31; 8.85)	47.77	0.84	-3.04	9.21	0.02494
miR-146b-3p	40.56 (37.63-44.23; 1.85)	40.40	41.26 (36.21-46.36; 2.89)	41.76	0.98	-0.93	0.87	0.47544
miR-146b-5p	77.89 (58.08-119.51; 15.1)	74.84	84.33 (57.54-118.32; 13.54)	83.36	0.92	-1.52	2.30	0.24627
miR-148a	39.47 (29.14-52.78; 7.4)	38.14	43.31 (30.19-67.51; 9.28)	42.63	0.91	-1.54	2.36	0.24108
miR-148b	263.41 (96.03-567.52; 113.34)	231.73	269.69 (96.59-519.93; 92.35)	254.25	0.98	-0.21	0.04	0.88123
*miR-149**	90.94 (61.9-140; 18.29)	93.77	100.14 (78.36-164.44; 19.28)	95.28	0.91	-1.65	2.73	0.20600
miR-150	735.29 (280.28-1464.34; 313.36)	650.89	803.25 (230.31-1299.92; 279.54)	805.43	0.92	-0.77	0.60	0.55262
miR-151-3p	90.5 (59.4-168.52; 28.65)	86.14	93.54 (40.3-200.16; 30.14)	90.27	0.97	-0.35	0.12	0.79773
miR-151-5p	400.24 (226.62-648.17; 119.05)	378.48	406.39 (171.5-713.97; 121.26)	395.05	0.98	-0.17	0.03	0.90478
miR-152	43.84 (34.82-51.15; 4.5)	44.10	42.3 (33.21-49.49; 4.23)	42.23	1.04	1.18	1.38	0.37332
miR-155	39.32 (33.4-51.97; 3.93)	38.49	40.2 (29.33-46.21; 4.14)	41.23	0.98	-0.72	0.52	0.58033
miR-15a	1067.1 (340.91-2264.86; 500.02)	926.59	1899.57 (342.45-3870.18; 859.99)	1754.90	0.56	-3.97	15.79	0.00485
miR-15b	5590.55 (2390.21-9314.28; 1631.47)	5449.72	6019.54 (2574.88-9330.56; 1614.39)	6022.98	0.93	-0.89	0.80	0.49652
*miR-15b**	33.4 (28.36-44.85; 3.99)	32.47	32.67 (26.32-38.49; 3.2)	32.96	1.02	0.67	0.45	0.61074
miR-16	9350.83 (5532-15791.68; 3088.31)	8363.37	12106.1 (3688.55-18634.11; 4135.14)	11851.02	0.77	-2.54	6.46	0.05538
miR-16-2*	52.16 (33.35-100.89; 19.86)	46.53	80.97 (31.06-148.04; 30.92)	77.93	0.64	-3.72	13.87	0.00723
miR-17	604.37 (252.59-1550.46; 343.17)	482.30	975.14 (277.42-2185.08; 443.34)	949.66	0.62	-3.15	9.91	0.02040
*miR-17**	68.17 (20.71-161.52; 40.51)	59.20	91.04 (25.25-230.66; 50.71)	87.40	0.75	-1.68	2.82	0.20052
miR-181a	53.14 (31.48-99; 20.27)	47.48	76.88 (36.28-154.59; 31.38)	72.12	0.69	-3.02	9.11	0.02518
*miR-181a-2**	32.53 (21.65-47.53; 7.81)	29.76	35.18 (20.88-48.68; 7.25)	36.21	0.92	-1.18	1.40	0.37332
miR-182	748.88 (430.36-1229.35; 200.08)	735.12	902.32 (295.83-1902.8; 405.28)	815.33	0.83	-1.61	2.59	0.21913
miR-1827	213.44 (155.58-436.62; 66.36)	189.74	240.91 (159.7-362.15; 61.51)	227.54	0.89	-1.45	2.10	0.26887
miR-183	119.68 (40.28-242.14; 49.38)	115.06	154.67 (51.98-367.62; 74.16)	146.40	0.77	-1.87	3.49	0.15787
*miR-183**	50.02 (36.15-61.65; 5.83)	49.18	52.36 (40.23-83.81; 9.45)	50.56	0.96	-1.00	0.99	0.45160
miR-185	1942.79 (787.89-5496.49; 1022.05)	1622.81	2373.4 (672.84-5393.38; 1062.43)	2105.79	0.82	-1.39	1.94	0.28649
*miR-185**	241.41 (98.14-620.84; 139.76)	212.41	292.11 (144.08-506.45; 92.98)	277.21	0.83	-1.45	2.10	0.26887
miR-186	115.21 (69.66-255.04; 42.45)	102.10	136.31 (47.39-219.81; 34.91)	133.33	0.85	-1.84	3.37	0.16334
*miR-187**	34.63 (29.31-39.69; 3.49)	34.62	33.26 (27.47-44.58; 4.01)	32.95	1.04	1.21	1.46	0.36312
miR-18a	103.44 (35.59-242.25; 62.86)	75.79	175.58 (37.45-477.68; 100.54)	160.15	0.59	-2.89	8.36	0.03129
*miR-18a**	202.6 (99.22-376.27; 58.98)	201.69	223.23 (57.16-406.03; 74.66)	223.81	0.91	-1.03	1.07	0.43916
miR-18b	44.08 (20.02-108.5; 24.7)	34.60	69.15 (20.79-181.32; 38.45)	62.24	0.64	-2.61	6.79	0.04986
miR-18b*	31.21 (27.51-36.64; 2.4)	31.10	37.26 (27.7-55.93; 6.15)	37.18	0.84	-4.29	18.43	0.00219
miR-1908	543.57 (240.09-1054.62; 213.26)	482.58	609.99 (314.34-1020.3; 175.86)	571.93	0.89	-1.15	1.32	0.38507
miR-190b	35.22 (32.94-37.72; 1.39)	35.32	32.61 (30.68-34.92; 1.06)	32.54	1.08	6.25	39.04	0.00002
miR-191	8469.29 (5040.96-11244.11; 1765.16)	8332.68	9594.15 (5347.3-14383.94; 2061.03)	9701.00	0.88	-1.98	3.90	0.13207
miR-1913	51.42 (43.36-55.63; 2.72)	51.63	52.33 (41.31-59.04; 4.21)	53.20	0.98	-0.85	0.72	0.51708
miR-192	106.77 (56.28-315.86; 54.58)	97.28	115.28 (46.97-181.2; 28.96)	115.88	0.93	-0.66	0.44	0.61594
miR-193a-3p	51.5 (32.01-79.34; 12.3)	48.58	54.18 (37.75-77.79; 9.6)	53.68	0.95	-0.82	0.68	0.52825
miR-193a-5p	35.59 (31.7-38.64; 1.63)	35.37	37.51 (32.21-52.9; 4.3)	36.69	0.95	-1.93	3.74	0.14175
*miR-193b**	34.52 (31.07-37.83; 1.71)	34.22	35.48 (31.02-39.31; 2.04)	35.88	0.97	-1.62	2.64	0.21408

miR-194	211.98 (110.23-782.51; 139.76)	183.22	210.31 (76.21-370.26; 63.36)	203.30	1.01	0.05	<0.01	0.96803
miR-195	156.44 (81.92-356.87; 63.44)	151.11	242 (66.42-421.8; 93.21)	231.30	0.65	-3.61	13.02	0.00894
*miR-196a**	49.33 (25.6-85.37; 14.88)	48.13	48.2 (25.9-68.94; 12.06)	48.52	1.02	0.28	0.08	0.84721
miR-197	100.78 (63.02-167.05; 27.37)	96.90	119.43 (55.68-185.85; 28.31)	121.81	0.84	-2.26	5.11	0.08922
miR-1973	36.44 (30.25-49.1; 4)	35.91	34.83 (30.03-46.45; 3.5)	34.27	1.05	1.43	2.04	0.27616
miR-1976	1141.76 (236.28-2384.41; 601.13)	1124.27	1936.79 (558.16-3532.59; 853.76)	2021.05	0.59	-3.62	13.12	0.00883
miR-198	43.97 (36.06-52.67; 4.63)	44.84	41.24 (33.53-50.44; 4.57)	40.46	1.07	1.98	3.94	0.13082
miR-199a-3p/miR-199b-3p	34.4 (24.65-49.18; 6.04)	33.89	32.79 (24.31-44; 5.4)	33.31	1.05	0.95	0.90	0.46685
miR-199a-5p	70.03 (34.53-143.41; 32.62)	60.56	57.65 (33.66-105.25; 15.63)	55.94	1.21	1.65	2.71	0.20650
miR-199b-5p	34.92 (28.01-42.35; 3.77)	34.96	32.85 (26.97-39.57; 3.13)	32.66	1.06	1.98	3.93	0.13082
miR-19a	346.21 (116-766.8; 193.68)	284.69	321.72 (93.59-636.05; 117.85)	323.04	1.08	0.52	0.27	0.69635
miR-19b	2353.74 (593.58-5971.5; 1616.42)	1762.68	1701.85 (219.95-2887.16; 681.78)	1692.05	1.38	1.79	3.20	0.17512
miR-200a	38.12 (27.89-52.96; 6.36)	37.81	33.51 (26.81-47.5; 5.14)	33.24	1.14	2.68	7.18	0.04431
miR-200b	37.01 (29.38-51.8; 5.31)	35.50	35.71 (28.28-50.68; 5.99)	34.59	1.04	0.77	0.60	0.55262
miR-200c	35.06 (26.34-51.49; 6.29)	33.30	33.36 (26.53-48.72; 5.56)	33.10	1.05	0.96	0.93	0.46152
miR-202	32.3 (29.57-35.09; 1.68)	32.25	33.39 (30.92-38.76; 1.76)	33.17	0.97	-1.99	3.95	0.13082
*miR-202**	48.72 (36.82-61.26; 7.47)	48.19	43.55 (34.19-55.59; 6.26)	42.07	1.12	2.53	6.38	0.05688
miR-204	42.91 (36.59-46.66; 2.93)	43.45	41.43 (33.48-47.02; 3.36)	41.56	1.04	1.55	2.41	0.23758
miR-205	53.05 (36.12-83.42; 11.64)	50.38	46.51 (33.62-77.42; 11.15)	44.62	1.14	1.94	3.75	0.14175
miR-205*	78.52 (61.61-99.99; 10.31)	76.54	69.12 (60.91-86.86; 6.23)	69.58	1.14	3.74	13.96	0.00723
miR-206	41.83 (34.46-55.24; 5.59)	42.47	39.42 (31.96-51.61; 4.89)	39.32	1.06	1.54	2.38	0.24108
miR-20a	315.49 (117.64-1009.48; 215.14)	275.17	560.07 (136.77-1413.7; 295.46)	540.34	0.56	-3.18	10.14	0.01914
miR-20b	50.43 (27.11-109.55; 24.24)	38.99	83.2 (26.11-188.58; 43.62)	79.55	0.61	-3.12	9.71	0.02144
miR-21	91.51 (59.81-147.09; 24.73)	86.92	119.91 (77.62-228.72; 34.12)	116.85	0.76	-3.21	10.28	0.01864
miR-210	48.32 (26.27-95.81; 18.76)	42.74	67.9 (29.69-134.86; 27.35)	66.01	0.71	-2.81	7.87	0.03579
*miR-2115**	34.13 (28.18-52.73; 4.94)	32.90	35.44 (28.86-40.8; 3.15)	36.51	0.96	-1.05	1.11	0.42970
miR-2116	56.31 (46.9-67.25; 6.02)	56.44	48.97 (41.9-52.94; 2.97)	50.01	1.15	5.21	27.15	0.00033
miR-214	37.8 (28.95-56.65; 6.56)	37.79	35.59 (28.01-50.46; 5.83)	35.05	1.06	1.20	1.43	0.36934
miR-215	136.77 (74.45-374.23; 61.04)	133.02	139.83 (61.64-214.52; 30.67)	140.20	0.98	-0.22	0.05	0.87769
miR-22	7276.87 (3091.68-18323.76; 3745.44)	6262.72	7182.75 (2684.6-12895.15; 2889.49)	6990.67	1.01	0.10	0.01	0.93836
*miR-22**	75.98 (57.37-105.85; 12.34)	75.38	75.51 (50.69-104.51; 11.74)	74.31	1.01	0.13	0.02	0.92430
miR-221	51.62 (44.76-70.74; 6.36)	50.22	58.52 (44.98-75.54; 8.34)	58.14	0.88	-3.12	9.71	0.02144
miR-222	95.81 (69.24-138.59; 18.54)	94.46	102.29 (74.72-133.38; 14.21)	102.28	0.94	-1.33	1.76	0.31338
miR-223	7440.93 (3212.02-13971.51; 3319.86)	6021.86	6982.53 (1514.29-12955.3; 2420.13)	6797.33	1.07	0.53	0.29	0.68737
miR-223*	34.13 (32.02-37.22; 1.36)	33.81	31.38 (28.79-34.18; 1.63)	31.03	1.09	5.63	31.71	0.00012
miR-224	33.46 (29.07-37.22; 2.25)	33.78	35.13 (31.08-42.57; 2.82)	34.94	0.95	-2.13	4.53	0.10696
miR-23a	609.65 (262.95-1325.85; 263.28)	586.22	537.8 (229.55-828.2; 133.51)	544.86	1.13	1.17	1.37	0.37417
miR-23b	946.92 (467.95-2213.02; 385.5)	873.79	792.5 (284.59-1089.12; 197.63)	861.45	1.19	1.71	2.94	0.19155
miR-24	630.38 (406.32-1208.5; 186.49)	618.64	658.87 (247.3-1066.18; 206.21)	665.90	0.96	-0.49	0.24	0.71338
*miR-24-1**	81.2 (54.91-122.43; 15.41)	80.06	85.3 (51.49-116.88; 15.93)	87.45	0.95	-0.88	0.78	0.49929
miR-25	1170.76 (668.03-1673.73; 284.65)	1207.38	1339.17 (408.86-2255.76; 428.24)	1338.84	0.87	-1.56	2.42	0.23658
*miR-25**	42.03 (33.97-60.88; 5.73)	40.34	40.56 (29.67-46.91; 3.62)	40.60	1.04	1.03	1.07	0.43916
miR-26a	1458.93 (845.47-2090.32; 364.27)	1535.26	1585.4 (504.06-2517.06; 406.7)	1645.25	0.92	-1.10	1.22	0.40607

miR-26b	313.57 (187.81-524.53; 77.27)	305.56	453.61 (143.59-949.03; 169.17)	447.29	0.69	-3.57	12.73	0.00952
*miR-26b**	32.56 (27.94-42.97; 3.24)	31.53	31.77 (26.99-35.52; 2.32)	31.89	1.02	0.92	0.85	0.48208
miR-27a	65.58 (47.25-87.86; 12.5)	62.39	63 (51.75-76.8; 8.51)	59.18	1.04	0.82	0.66	0.53175
miR-27b	50.85 (37.94-71.4; 9.92)	47.94	50.26 (39.33-63.11; 6.82)	50.26	1.01	0.23	0.05	0.86965
miR-28-5p	59.1 (31.78-91.25; 18.18)	56.04	70.55 (30.04-137.61; 23.31)	70.54	0.84	-1.84	3.40	0.16209
miR-296-5p	53.3 (45.37-58.75; 3.54)	54.05	51.88 (41.75-57.28; 4.42)	52.97	1.03	1.18	1.38	0.37332
miR-299-3p	27.16 (14.03-67.05; 12.62)	23.48	33.4 (13.79-89.69; 18.21)	29.95	0.81	-1.34	1.79	0.31150
miR-29a	144.68 (66.18-296.22; 64.21)	119.59	133.66 (78.24-214.91; 39)	125.90	1.08	0.70	0.50	0.59085
miR-29b	316.63 (131.77-811.82; 170.81)	237.25	301.13 (85.02-510.23; 116.04)	292.87	1.05	0.36	0.13	0.79396
*miR-29b-2**	28.03 (20.04-55.47; 9.67)	23.99	30.88 (19.42-46.36; 7.77)	31.49	0.91	-1.10	1.20	0.41054
miR-29c	66.04 (36.13-119.4; 24.99)	55.44	70.65 (44.42-128.64; 19.21)	64.41	0.93	-0.70	0.49	0.59198
*miR-29c**	42.32 (29.96-71.12; 8.84)	41.23	41.15 (25.62-53.27; 6.45)	41.36	1.03	0.51	0.26	0.69967
miR-300	61.52 (47.13-82.98; 7.64)	60.35	57.84 (50.62-63.95; 4.08)	58.60	1.06	2.03	4.12	0.12187
miR-301a	29.15 (21.07-49.39; 8.3)	24.37	38.24 (20.16-83.08; 13.51)	37.16	0.76	-2.72	7.39	0.04194
miR-30a	89.3 (54.24-160.58; 24.62)	91.64	94.33 (56.34-132.58; 21.67)	95.63	0.95	-0.73	0.54	0.57660
miR-30b	3296.72 (1194.87-5857.69; 1321.74)	2764.15	2521.33 (532.29-3446.48; 693.09)	2700.82	1.31	2.50	6.24	0.06005
miR-30c	4089.42 (1333.93-6525.43; 1508.41)	3895.46	3584.19 (1065.98-5419.78; 964.07)	3610.72	1.14	1.35	1.83	0.30338
miR-30d	437.61 (219.5-802.18; 137.83)	413.86	484.48 (245.09-706.42; 107.18)	487.67	0.90	-1.29	1.65	0.33125
miR-30e	238.74 (112.97-503.87; 107.29)	198.36	272.28 (93.17-529.48; 101.74)	265.21	0.88	-1.08	1.17	0.41627
*miR-30e**	141.21 (63.39-251.71; 46.51)	133.09	129.7 (46.49-214.77; 33.27)	133.16	1.09	0.96	0.93	0.46152
miR-32*	146.41 (120.49-182.44; 16.45)	145.91	131.49 (103.67-161.5; 14.29)	130.39	1.11	3.27	10.70	0.01673
miR-3202	49.26 (37.05-74.44; 9.09)	47.07	41.74 (30.77-72.95; 9.08)	40.71	1.18	2.78	7.75	0.03697
miR-320a	217.02 (125.36-456.81; 84.03)	188.20	335.37 (163.66-875.64; 171.35)	322.10	0.65	-2.94	8.65	0.02875
miR-320b	200.87 (120.64-410.83; 73.78)	174.04	310.47 (155.81-799.47; 155.7)	289.21	0.65	-3.02	9.09	0.02518
miR-320c	207.32 (122.87-434.67; 83.05)	178.33	339.03 (159.1-856.97; 172.55)	321.27	0.61	-3.26	10.63	0.01692
miR-320d	159.6 (97.62-325.91; 58.81)	139.99	242.34 (126.94-615.37; 118.19)	220.87	0.66	-2.97	8.83	0.02689
miR-324-3p	63.75 (33.8-92.82; 18.54)	61.62	78.09 (37.44-110.54; 20.99)	81.88	0.82	-2.44	5.96	0.06586
miR-324-5p	69.34 (39.32-116.15; 20.9)	62.12	87.85 (51.96-130.58; 22.54)	82.72	0.79	-2.87	8.25	0.03163
miR-326	513.77 (102.2-933.29; 261.23)	531.90	739.33 (266.07-1406.34; 301.85)	754.32	0.69	-2.69	7.26	0.04327
miR-328	85.43 (60.67-106.38; 13.43)	84.22	91.53 (61.64-156.84; 18.01)	88.36	0.93	-1.29	1.67	0.33031
miR-331-3p	335.39 (169.47-797.4; 136.02)	288.06	326.27 (147.65-531.13; 77.05)	328.39	1.03	0.28	0.08	0.84721
miR-335	91.24 (40.82-196.65; 31.01)	87.45	88.03 (57.91-150.83; 22.28)	82.05	1.04	0.40	0.16	0.76833
*miR-335**	590.09 (348.13-940.25; 164.72)	561.98	507.73 (248.76-1269.62; 218.83)	434.67	1.16	1.43	2.05	0.27506
miR-338-3p	33.5 (29.84-36.63; 1.73)	33.36	31.98 (26.72-35.44; 2.41)	31.87	1.05	2.31	5.35	0.08032
miR-339-3p	71.23 (28.53-138.29; 29.61)	69.89	78.22 (30.82-133.16; 26.04)	76.19	0.91	-0.85	0.72	0.51708
miR-339-5p	455.77 (122.17-891.72; 215.98)	436.32	332.27 (90.23-581.56; 114.96)	309.35	1.37	2.43	5.89	0.06770
miR-340	36.89 (30.98-43.11; 3.38)	35.68	38.65 (33.88-52.4; 4.26)	37.49	0.95	-1.52	2.30	0.24627
*miR-340**	37.61 (27.26-62.09; 10.06)	34.76	38.72 (22.67-55.79; 7.24)	37.82	0.97	-0.43	0.18	0.75212
miR-342-3p	274.07 (135.07-478.59; 87.57)	262.39	305.57 (154.7-434.31; 84.4)	304.84	0.90	-1.24	1.53	0.34983
miR-34a	46.89 (30.86-68.24; 8.98)	46.53	42.59 (34.28-61.57; 7.68)	42.61	1.10	1.74	3.02	0.18659
miR-34b	227.13 (127.64-412.72; 84.69)	210.57	216.68 (132.47-323.35; 62.02)	216.35	1.05	0.48	0.23	0.71877
miR-361-3p	90.75 (75.52-103.75; 6.14)	90.08	91.98 (79.27-120.93; 9.66)	90.26	0.99	-0.51	0.26	0.70230
miR-361-5p	79.36 (58.38-118.65; 14.83)	76.79	80.79 (55.04-94.84; 11.04)	84.01	0.98	-0.37	0.14	0.78955

miR-362-5p	49.42 (33.89-77.84; 13.25)	42.37	56.74 (29.98-99.82; 17.18)	56.37	0.87	-1.60	2.57	0.21963
miR-363	595.42 (351.54-994.23; 207.56)	544.44	694.76 (137.65-1204.01; 270.2)	681.56	0.86	-1.39	1.93	0.28837
*miR-363**	37.11 (30.38-45.46; 3.84)	35.96	34.8 (30.4-48.94; 3.55)	34.16	1.07	2.08	4.31	0.11483
miR-365	85.94 (62.07-114.36; 14.48)	86.16	89.68 (70.95-129.9; 15.13)	89.72	0.96	-0.85	0.73	0.51650
miR-374a	89.73 (29.86-206.87; 49.73)	71.93	102.58 (24.17-227.24; 48.38)	104.94	0.87	-0.88	0.78	0.49929
miR-374b/miR-374c	77.05 (25.51-178.06; 36.46)	69.47	112.17 (22.36-239.06; 46.7)	111.14	0.69	-2.82	7.96	0.03472
miR-378/miR-378c	113.47 (74.1-183.99; 32)	105.53	124.09 (75.84-216.78; 31.29)	122.19	0.91	-1.13	1.28	0.39434
miR-384	30.68 (20.41-50.06; 8.86)	26.61	27.72 (18.66-36.1; 4.63)	29.02	1.11	1.42	2.00	0.27698
miR-3943	32.64 (29.03-35.33; 1.86)	32.81	32.2 (28.03-42.95; 2.88)	32.06	1.01	0.59	0.34	0.66055
miR-423-3p	424.63 (267.56-869.43; 140.6)	407.62	358.81 (210.85-529.87; 71.87)	362.46	1.18	2.00	4.02	0.12723
miR-423-5p	522.66 (307.27-909.7; 171.37)	496.31	708.75 (380.77-1316.36; 256.12)	643.07	0.74	-2.87	8.24	0.03163
miR-424	57.61 (35.16-81.66; 11.51)	56.47	57.53 (43.71-76.87; 9.38)	56.16	1.00	0.03	<0.01	0.98184
miR-425	488.29 (168.86-801.87; 183.71)	450.16	595.5 (126.33-1164.18; 242.18)	628.89	0.82	-1.68	2.82	0.20052
*miR-425**	31.44 (23.91-43.11; 5.94)	29.97	35.64 (23.66-50.03; 7.67)	35.20	0.88	-2.06	4.23	0.11617
miR-450a	39.22 (33.96-42.85; 2.26)	39.67	36.33 (32.92-43.47; 2.23)	36.10	1.08	4.16	17.31	0.00298
miR-451	32594.25 (20177.97-47418.38; 6698.16)	34768.30	31812.88 (19324.36-45081.27; 7180.75)	31452.67	1.02	0.38	0.14	0.78300
miR-483-3p	140.68 (98.26-225.28; 32.16)	132.83	139.15 (97.51-201.9; 28.04)	141.38	1.01	0.17	0.03	0.90478
miR-483-5p	44.92 (35.77-52.44; 4.69)	45.23	41.05 (32.08-53.72; 5.27)	39.80	1.09	2.59	6.71	0.05024
miR-484	590 (393.99-827.24; 117.27)	567.97	642.24 (362.38-994.5; 146.73)	662.63	0.92	-1.32	1.75	0.31414
miR-485-3p	39.81 (34.82-45.76; 2.8)	39.77	40.73 (33.5-47.11; 3.99)	41.83	0.98	-0.88	0.78	0.49929
miR-485-5p	35.07 (32.31-37.65; 1.38)	34.93	33.67 (30.8-38.82; 2.08)	33.42	1.04	2.49	6.20	0.06005
miR-486-5p	7571.66 (4342.52-10243.01; 1721.88)	8192.17	7904.49 (4498.4-11401.37; 1740.28)	7739.88	0.96	-0.65	0.42	0.62317
miR-491-3p	43.71 (33.05-56.36; 7.09)	44.53	43.41 (27.91-60.75; 7.51)	42.91	1.01	0.14	0.02	0.92430
miR-494	182.07 (107.57-302.03; 43.97)	177.17	137.38 (108.4-174.96; 17.39)	136.08	1.33	4.55	20.71	0.00134
miR-498	57.03 (44.7-79.55; 7.73)	56.29	58.47 (48.33-79.28; 7.58)	58.12	0.98	-0.63	0.40	0.63332
miR-500a	39.64 (28.68-59.25; 8.25)	36.25	44.31 (30.47-77.32; 10.42)	44.72	0.89	-1.67	2.79	0.20279
*miR-500a**	66.72 (36.64-117.77; 22.33)	61.97	82.32 (35.03-174.96; 31.96)	80.47	0.81	-1.90	3.62	0.14895
miR-501-5p	30.99 (24.69-42.1; 5.04)	29.68	36.5 (24.45-62.25; 8.55)	36.49	0.85	-2.62	6.88	0.04934
miR-502-3p	59.71 (22.05-119.71; 29.72)	50.72	78.51 (25.1-179.84; 35.92)	77.30	0.76	-1.92	3.69	0.14510
miR-505	37.17 (26.44-64.19; 9.07)	36.10	35.86 (23.07-50.86; 6.4)	33.47	1.04	0.56	0.32	0.66938
*miR-505**	75.45 (63.31-92.92; 8.02)	72.67	78.43 (55.2-92.52; 8.16)	79.72	0.96	-1.24	1.53	0.34983
miR-508-3p	32.04 (29.63-34.56; 1.36)	31.90	31.18 (27.68-33.76; 1.6)	31.50	1.03	1.79	3.21	0.17512
miR-513a-5p	129.68 (82.43-164.59; 23.18)	132.58	118.84 (81.65-179.71; 20.4)	114.74	1.09	1.68	2.82	0.20052
miR-513c	32.82 (30.12-35.79; 1.42)	33.14	31.95 (28.53-37.57; 2.28)	31.59	1.03	1.45	2.11	0.26887
miR-516a-5p	42.63 (33.76-50.66; 4.2)	41.94	37.71 (28.38-73.77; 8.95)	35.31	1.13	2.35	5.51	0.07578
miR-516b	37.76 (29.74-51.89; 5.9)	36.89	33.87 (26.6-47.34; 4.95)	34.09	1.11	2.40	5.75	0.07074
miR-517b	43.27 (38.07-47.63; 3.27)	42.79	39.87 (35.32-44.96; 3.23)	39.36	1.09	3.46	12.00	0.01196
miR-517c	40.2 (35.27-48.88; 3.1)	39.97	36.41 (32.48-39.57; 2)	36.69	1.10	4.77	22.74	0.00095
miR-518b	35.04 (27.06-51.27; 5.66)	34.14	35.91 (28.73-52.88; 5.61)	35.28	0.98	-0.52	0.27	0.69635
miR-518e/miR-519a*/miR-519b-5p/miR-519c-5p/miR-522*/miR-523**	37.97 (32.1-45.48; 4.01)	37.68	35.83 (28.37-50.83; 5.57)	35.15	1.06	1.47	2.15	0.26478
miR-519b-3p	35.43 (23.03-55.75; 7.64)	34.66	33.59 (23.5-54.35; 8.01)	32.42	1.05	0.79	0.62	0.54625

miR-519d	48.58 (39.63-58.4; 5.28)	49.14	46.11 (38.62-67.2; 6.33)	44.21	1.05	1.42	2.01	0.27698
*miR-519e**	36.18 (31.64-42.13; 2.85)	36.21	35.3 (30.02-41.87; 2.93)	35.38	1.02	1.00	1.00	0.45122
miR-520d-5p	37.39 (27.09-52.81; 6.43)	35.71	42.16 (32.98-51.51; 5.85)	42.22	0.89	-2.61	6.80	0.04986
miR-521	32.19 (23.71-42.74; 4.93)	31.82	30.36 (23.02-42.1; 4.85)	29.72	1.06	1.25	1.57	0.34646
miR-522	32.5 (27.4-41.65; 2.77)	31.90	34.26 (27.72-40.21; 3.53)	33.37	0.95	-1.83	3.33	0.16636
miR-525-5p	43.25 (33.2-55.38; 5.3)	41.76	37.7 (30.18-53.73; 5.68)	36.63	1.15	3.38	11.45	0.01409
miR-532-3p	86.11 (46.41-121.81; 16.41)	86.97	95.6 (44.97-144.99; 24.84)	92.76	0.90	-1.51	2.29	0.24647
miR-532-5p	48.21 (23.64-89.63; 18.57)	42.22	64.83 (24.7-123.24; 26.38)	67.31	0.74	-2.45	6.00	0.06518
miR-550a	41.86 (25.35-76.03; 13.44)	38.08	49.34 (24.95-81.45; 14.71)	49.28	0.85	-1.79	3.20	0.17512
*miR-550a**	149.38 (62.32-322.87; 67.74)	127.71	160.26 (38.85-287.53; 53.33)	163.27	0.93	-0.60	0.36	0.64782
miR-551a	33.03 (30.41-36.03; 1.59)	33.05	33.69 (28.64-39.72; 2.28)	33.72	0.98	-1.06	1.13	0.42557
miR-551b	493.03 (254.67-928.98; 188.43)	455.32	406.77 (262.58-704.83; 108.71)	379.53	1.21	1.90	3.63	0.14895
miR-574-3p	79.42 (59.99-105.52; 12.13)	79.51	88.46 (73.45-105.57; 7.82)	88.47	0.90	-3.00	9.00	0.02593
miR-574-5p	106.21 (91.36-137.16; 12.03)	105.88	96.12 (78.93-115.4; 8.94)	93.90	1.11	3.22	10.37	0.01828
miR-576-5p	186.31 (78.52-335.35; 82.1)	165.63	197.86 (39.2-298.39; 63.23)	213.27	0.94	-0.53	0.28	0.68737
miR-583	58.13 (46.07-75.54; 6.97)	58.71	52.64 (41.89-70.85; 6.88)	52.63	1.10	2.66	7.10	0.04574
miR-584	45.24 (27.73-73.07; 13.95)	42.57	59.22 (25.76-128.76; 22.72)	57.99	0.76	-2.49	6.20	0.06005
miR-585	32.63 (29.07-37.92; 2.49)	31.62	31.09 (27.43-38.8; 2.41)	31.21	1.05	2.05	4.22	0.11617
miR-593	36.15 (32.74-39.46; 1.49)	36.34	34.42 (31.48-37.73; 1.78)	33.85	1.05	3.29	10.80	0.01639
*miR-593**	34.25 (22.88-48.72; 7.8)	32.81	36.84 (23.56-54.53; 8.29)	36.72	0.93	-1.08	1.17	0.41627
miR-601	35.59 (32.05-40.88; 2.22)	35.40	32.95 (29.69-35.08; 1.28)	32.89	1.08	4.64	21.49	0.00131
miR-602	49.47 (42.31-71.53; 6.8)	49.17	48.44 (35.52-57.44; 5.03)	48.50	1.02	0.58	0.34	0.66140
miR-615-5p	35.56 (32.18-39.26; 2.07)	35.79	35.03 (29.9-42.71; 2.64)	35.20	1.02	0.72	0.52	0.58033
miR-617	34.69 (32.18-38.6; 1.9)	34.90	33.38 (30.77-39.39; 1.87)	33.23	1.04	2.22	4.91	0.09291
miR-618	34.52 (30.79-38.91; 1.88)	34.42	32.96 (29.9-38.24; 2.34)	32.35	1.05	2.36	5.57	0.07474
miR-620	62.23 (48.08-73.8; 7.45)	62.87	53.58 (43.81-68.31; 5.24)	53.31	1.16	4.53	20.48	0.00134
miR-622	37.8 (34.17-40.92; 1.71)	37.85	37.21 (32.37-44.42; 2.82)	37.04	1.02	0.81	0.66	0.53345
miR-625	35.51 (21.49-79.03; 13.2)	31.95	39.95 (22.91-75.49; 11.52)	38.79	0.89	-1.21	1.47	0.36312
*miR-625**	85.61 (60.31-119.06; 14.99)	80.90	81.23 (65.02-136.28; 15.11)	78.67	1.05	0.98	0.96	0.45633
miR-628-3p	126.01 (78.69-286.9; 48.45)	107.80	130.31 (82.83-197.68; 31.1)	124.39	0.97	-0.36	0.13	0.79396
miR-629	40.63 (28.96-66.23; 8.41)	39.35	47.03 (27.56-67.77; 10.62)	46.13	0.86	-2.24	5.03	0.09028
miR-630	41.1 (37.36-45.42; 2.43)	41.37	38.6 (36.32-42.48; 1.7)	38.52	1.06	3.84	14.76	0.00564
miR-634	548.59 (190.18-1227.06; 274.62)	496.03	749.81 (378.35-1229.78; 247.68)	759.46	0.73	-2.60	6.77	0.04992
miR-636	35.4 (29.32-47.66; 4.01)	34.88	36.51 (26.5-43.14; 3.61)	36.25	0.97	-0.97	0.94	0.46135
miR-637	114.68 (55.84-265.41; 56.77)	97.56	131.64 (78.1-231.21; 39.99)	122.62	0.87	-1.17	1.37	0.37417
miR-638	62.31 (49.09-82.34; 7.85)	60.12	60.75 (51.21-78.87; 7.69)	58.07	1.03	0.68	0.46	0.60925
miR-642a	118.26 (86.97-163.33; 17.49)	119.26	119.4 (95.95-149.96; 13.8)	115.90	0.99	-0.24	0.06	0.86431
miR-652	126.41 (49.51-267.15; 53.51)	123.52	202.94 (63.08-372.45; 75.64)	211.63	0.62	-3.93	15.43	0.00493
miR-659	35.3 (32.9-38.38; 1.58)	34.84	34.33 (30.7-38.74; 1.93)	34.43	1.03	1.73	2.98	0.18753
miR-660	63.08 (36.93-109.95; 23.04)	50.90	73.83 (36.76-157.9; 27.84)	71.03	0.85	-1.42	2.01	0.27698
miR-664	79.04 (63.63-94.88; 7.7)	78.63	79.78 (70.86-91.72; 6.35)	79.98	0.99	-0.35	0.13	0.79470
miR-665	39.88 (31.29-59.02; 5.97)	38.73	38.89 (34.57-50.76; 4.35)	37.15	1.03	0.64	0.40	0.63024
miR-675	44.81 (35.1-55.31; 5.24)	44.63	40.19 (32.42-52.45; 5.59)	39.88	1.12	2.86	8.15	0.03217

*miR-675**	41.85 (36.76-47.97; 2.51)	41.54	41.22 (35.66-47.38; 2.79)	41.30	1.02	0.79	0.62	0.54725
miR-7	88.34 (64.35-153.78; 23.06)	81.46	105.46 (61.72-155.56; 25.23)	108.00	0.84	-2.39	5.71	0.07099
miR-718	44.84 (37.17-55.93; 3.93)	44.78	53.23 (37.84-121.01; 16.27)	49.41	0.84	-2.37	5.60	0.07438
miR-720	340.15 (163.4-579.45; 119.55)	342.21	252.95 (154.14-455.46; 74.57)	238.94	1.34	2.97	8.82	0.02689
miR-744	161.25 (111.13-280.29; 42.76)	145.36	178.97 (140.62-252.91; 28.83)	175.53	0.90	-1.65	2.71	0.20650
miR-765	98.55 (69.96-127.38; 15.92)	100.00	86.54 (58.64-119.37; 12.06)	86.25	1.14	2.88	8.28	0.03163
miR-766	57.61 (42.7-75.07; 7.83)	57.13	67.4 (38.23-91.64; 12.12)	68.59	0.85	-3.22	10.37	0.01828
miR-877	35.79 (31.12-41.23; 2.73)	35.87	34.29 (30.91-38.09; 1.95)	34.25	1.04	2.06	4.26	0.11617
*miR-877**	38.87 (36.02-44.1; 2.1)	38.32	39.93 (33.55-67.04; 6.38)	39.15	0.97	-0.74	0.55	0.57055
miR-885-5p	62.64 (52.85-71.2; 4.74)	62.01	70.19 (53.53-88.13; 7.58)	70.63	0.89	-3.99	15.92	0.00482
*miR-888**	101.04 (79.44-125.47; 14.66)	103.44	114.78 (86.19-186.09; 23.89)	108.08	0.88	-2.33	5.42	0.07831
miR-891a	29.83 (23.94-36.1; 4.26)	28.28	34.44 (23.51-68.6; 9.63)	32.21	0.87	-2.06	4.25	0.11617
miR-892b	33.42 (31.37-36.94; 1.66)	32.82	31.03 (28.73-35.5; 1.65)	30.56	1.08	4.50	20.27	0.00135
miR-92a	6137.21 (3682.25-7585.89; 961.75)	6273.77	6095.17 (4231.6-8086.36; 1110.34)	6142.59	1.01	0.14	0.02	0.92430
miR-92b	703.8 (403.24-1020.68; 150.33)	681.28	758.02 (279.07-1181.25; 213.18)	715.01	0.93	-0.99	0.98	0.45289
miR-93	585.97 (184.4-1562.58; 374.69)	439.86	980.62 (237.19-2015.51; 477.89)	1052.16	0.60	-3.09	9.58	0.02185
*miR-93**	89.08 (45.9-173.42; 27.39)	85.35	89.67 (28.61-122.09; 24.29)	95.73	0.99	-0.08	0.01	0.95151
miR-933	129.39 (85.96-194.74; 30.34)	129.56	119.38 (79.76-165.13; 26.81)	115.59	1.08	1.18	1.40	0.37332
miR-942	48.77 (41.92-57.79; 3.51)	48.84	43 (37.68-46.68; 2.31)	43.66	1.13	6.43	41.34	0.00002
miR-943	64.87 (49.74-74.07; 7.27)	65.37	65.14 (52.41-92.39; 9.19)	63.10	1.00	-0.11	0.01	0.92789
miR-96	80.53 (39.8-146.1; 28.03)	75.63	99.17 (40.58-218.65; 41.81)	91.83	0.81	-1.76	3.10	0.18095
miR-98	368.69 (135.63-1290.59; 291.6)	282.28	501.71 (162.8-969.23; 223.14)	512.12	0.73	-1.74	3.01	0.18659
miR-99a	48.33 (27-79.86; 12.69)	46.94	41.69 (26.72-54.7; 6.15)	40.98	1.16	2.26	5.10	0.08922
miR-99b	46.17 (30.56-62.6; 8.45)	44.59	45.16 (31.25-59.62; 6.21)	44.99	1.02	0.46	0.21	0.73036
miRPlus-A1015	175.57 (79.42-324.77; 73.53)	173.18	150.24 (77.98-243.75; 41.91)	141.68	1.17	1.44	2.07	0.27323
miRPlus-A1072	47.67 (32.93-59.09; 6.92)	48.74	46.12 (34.65-64.53; 7.03)	44.86	1.03	0.75	0.56	0.57037
miRPlus-A1073	51.09 (44.93-58.43; 2.94)	51.13	52.25 (44.25-63.3; 4.81)	51.19	0.98	-0.96	0.92	0.46181
miRPlus-C1110	312.42 (183.58-454.85; 62.17)	310.21	224.99 (139.41-329.64; 50.54)	218.01	1.39	5.22	27.28	0.00033
miRPlus-D1058	31.26 (22.13-54; 8.61)	29.00	40.78 (25.47-65.25; 10.33)	41.19	0.77	-3.37	11.34	0.01442
miRPlus-E1012	31.98 (26.46-39.78; 3.32)	31.29	29.56 (24.11-36.6; 2.78)	29.16	1.08	2.62	6.85	0.04948
miRPlus-E1016	211.82 (59.51-588.24; 129.47)	164.06	133.2 (66.45-258.68; 53.13)	115.51	1.59	2.71	7.32	0.04245
miRPlus-E1031	61.17 (46.76-92.78; 10.9)	58.87	56.02 (46.02-64.39; 4.98)	57.03	1.09	2.06	4.24	0.11617
miRPlus-E1033	230.39 (120.79-711.48; 116.18)	206.21	198.75 (101.87-293.61; 54.48)	195.39	1.16	1.19	1.41	0.37332
miRPlus-E1038	193.66 (112.02-328.44; 56.92)	201.24	154.53 (76.94-258.21; 50.76)	142.30	1.25	2.45	6.02	0.06511
miRPlus-E1072	31.42 (25.79-39.78; 3.83)	31.48	31.28 (23.16-38.38; 3.47)	31.18	1.00	0.12	0.02	0.92555
miRPlus-E1077	46.66 (41.76-51.82; 2.88)	46.93	44.81 (38.89-51.88; 3.41)	44.21	1.04	1.94	3.75	0.14175
miRPlus-E1082	35.8 (30.82-42.42; 3.47)	35.35	36.78 (26.78-46.24; 4.86)	35.57	0.97	-0.78	0.60	0.55262
miRPlus-E1088	506.53 (203.98-994.12; 212.32)	460.56	408.66 (210.03-642.28; 135.89)	400.09	1.24	1.86	3.47	0.15826
miRPlus-E1090	32.32 (28.22-36.94; 2.63)	32.32	29.29 (22.93-34.94; 2.46)	29.00	1.10	3.89	15.11	0.00534
miRPlus-E1093	47.65 (40.57-60.31; 5.26)	47.28	43.08 (36.38-52.72; 3.56)	42.43	1.11	3.41	11.65	0.01321
miRPlus-E1097	62.17 (50.41-74.71; 7.28)	62.67	54.98 (44.8-64.49; 4.73)	55.06	1.13	3.95	15.60	0.00493
miRPlus-E1098	59.69 (46.4-72.94; 6.6)	59.60	56.58 (47.54-72.05; 5.17)	56.74	1.05	1.77	3.12	0.18095
miRPlus-E1104	89.94 (62.17-129.63; 18.58)	90.04	78.57 (59.18-95.71; 10.15)	77.27	1.14	2.58	6.65	0.05089

miRPlus-E1110	50.69 (44.01-55.6; 2.84)	50.54	48.22 (44.61-54.27; 2.39)	48.32	1.05	3.08	9.49	0.02237
miRPlus-E1117	207.96 (112.29-483.11; 77.56)	199.65	193.08 (115.93-273.39; 39.39)	189.64	1.08	0.82	0.68	0.52825
miRPlus-E1146	98.79 (74.47-137.98; 13.12)	97.58	96.6 (79.66-119.84; 11.12)	95.62	1.02	0.61	0.37	0.64601
miRPlus-E1151	96.52 (70.13-118.65; 11.77)	96.40	81.91 (61.21-110.55; 10.5)	80.87	1.18	4.42	19.53	0.00165
miRPlus-E1153	66.12 (54.04-92.8; 9.18)	63.00	62.09 (47.43-74.82; 7.09)	63.01	1.06	1.66	2.74	0.20600
miRPlus-E1172	115.04 (79.41-178.31; 26.47)	113.44	113.58 (89.69-153.29; 16.65)	110.26	1.01	0.22	0.05	0.87288
miRPlus-E1175	42.18 (38.39-47.3; 2.08)	41.80	39.78 (35.47-43.26; 1.82)	39.85	1.06	3.93	15.42	0.00493
miRPlus-E1189	38.5 (31.73-45.99; 3.05)	37.95	40.27 (33.26-47.32; 3.64)	40.66	0.96	-1.74	3.04	0.18580
miRPlus-E1193	33.35 (29.85-38.9; 2.55)	32.88	33.67 (29.01-39.57; 2.56)	33.06	0.99	-0.41	0.17	0.76588
miRPlus-E1200	55.27 (48.16-64.5; 4.38)	54.56	56.64 (49.81-65.97; 3.8)	56.48	0.98	-1.12	1.25	0.40033
miRPlus-E1212	179.82 (143.32-226.27; 20.78)	183.28	155 (120.49-201.19; 17.76)	149.44	1.16	4.34	18.84	0.00199
miRPlus-E1225	82.21 (53.02-132.22; 19.25)	77.26	85.24 (51.95-113.85; 16.38)	86.52	0.96	-0.57	0.33	0.66343
miRPlus-E1232	59.67 (46.58-74.67; 7.25)	60.63	51.22 (43.09-58.74; 3.62)	51.41	1.17	4.98	24.79	0.00061
miRPlus-E1238	69.17 (55.78-93.1; 9.91)	68.87	67.73 (49.51-93.7; 10.15)	66.05	1.02	0.48	0.23	0.71543
miRPlus-E1245	66.4 (47.35-88.37; 12.79)	63.72	67.35 (51.4-93.46; 10.66)	64.47	0.99	-0.27	0.07	0.84834
miRPlus-E1247	98.47 (71.27-183.62; 26.37)	91.79	80.87 (65.58-92.76; 6.94)	80.83	1.22	3.11	9.69	0.02144
miRPlus-E1258	86.75 (56.01-125.13; 20.49)	83.05	64.07 (48.87-100.32; 11.77)	61.78	1.35	4.61	21.22	0.00131
miRPlus-E1285	71.73 (60.83-86.43; 7.24)	72.89	71.17 (54.83-93.29; 8.2)	69.16	1.01	0.24	0.06	0.86431
miRPlus-F1004	43.52 (39.06-47.3; 2.29)	43.65	43.55 (37.63-46.95; 2.57)	44.36	1.00	-0.05	<0.01	0.96857
miRPlus-F1014	32.91 (29.76-35.43; 1.54)	33.07	30.75 (28.5-33.5; 1.37)	30.61	1.07	4.54	20.59	0.00134
miRPlus-F1017	64.29 (47.23-88.15; 11.28)	60.60	59.48 (46.37-76.1; 7.79)	58.90	1.08	1.68	2.82	0.20052
miRPlus-F1023	49.66 (41.27-56.97; 3.46)	49.34	48.43 (43.29-63.07; 4.21)	47.55	1.03	1.06	1.12	0.42721
miRPlus-F1026	55.93 (40.21-66.98; 6.65)	55.13	59.96 (47.41-75.05; 6.12)	59.64	0.93	-2.12	4.49	0.10884
miRPlus-F1036	31.77 (26.2-36.35; 2.29)	32.09	32.54 (27.73-40.62; 3.46)	32.28	0.98	-0.85	0.73	0.51650
miRPlus-F1041	49.34 (41.84-58.66; 3.78)	49.98	46.95 (41.96-55.79; 3.24)	46.87	1.05	2.25	5.08	0.08941
miRPlus-F1042	54.62 (41.6-63.68; 5.55)	55.80	48.89 (36.28-66.54; 7.44)	47.99	1.12	2.92	8.54	0.02984
miRPlus-F1055	34.5 (31.21-38.36; 1.99)	34.64	32.67 (27.17-39.01; 2.49)	32.42	1.06	2.62	6.87	0.04934
miRPlus-F1058	47.26 (29.33-109.75; 20.04)	41.35	60.5 (29.02-107.04; 19.89)	62.91	0.78	-2.24	5.01	0.09028
miRPlus-F1062	42.89 (34.55-54.46; 3.76)	42.45	44.28 (34.61-52.26; 4.52)	44.92	0.97	-1.12	1.25	0.40033
miRPlus-F1064	79.67 (61.15-101.09; 10.28)	79.87	73.61 (61.88-105.49; 9.23)	73.29	1.08	2.09	4.38	0.11240
miRPlus-F1066	89.98 (71.55-118.02; 13.27)	90.38	82.46 (59.14-117.51; 10.4)	83.12	1.09	2.13	4.54	0.10696
miRPlus-F1074	109.08 (44.3-331.61; 70.85)	90.41	157.57 (48.96-348.08; 75.23)	146.70	0.69	-2.24	5.01	0.09028
miRPlus-F1080	34.99 (29.44-48.32; 4.37)	34.03	36.6 (27.78-41.07; 3.08)	37.02	0.96	-1.42	2.02	0.27698
miRPlus-F1086	45.61 (36.85-53.85; 4.84)	46.42	41.19 (35.59-52.28; 3.96)	39.73	1.11	3.35	11.22	0.01474
miRPlus-F1099	33.55 (29.51-37.58; 2.27)	33.69	34.49 (29.35-47.64; 3.68)	34.26	0.97	-1.01	1.02	0.44771
miRPlus-F1127	31.77 (29.01-34.05; 1.36)	31.85	33.42 (29.02-35.93; 1.7)	33.79	0.95	-3.29	10.83	0.01639
miRPlus-F1130	66.17 (57.6-71.07; 3.25)	66.73	63.21 (57.29-73.25; 4.13)	62.98	1.05	2.64	6.96	0.04823
miRPlus-F1147	60.23 (43.61-104.06; 15.25)	57.18	67.1 (47.61-106.71; 15.31)	64.76	0.90	-1.52	2.30	0.24627
miRPlus-F1153	35.71 (32.2-41.75; 2.28)	35.40	35.21 (27.49-42.12; 2.68)	35.81	1.01	0.65	0.42	0.62317
miRPlus-F1154	53.84 (42.97-63.91; 5.71)	54.75	58.33 (46.62-97.97; 11.23)	56.64	0.92	-1.68	2.84	0.20052
miRPlus-F1155	76.22 (69.56-82.32; 4.02)	76.50	75.76 (69.09-85.05; 3.81)	75.84	1.01	0.39	0.16	0.77318
miRPlus-F1162	36.18 (33.3-38.84; 1.8)	36.35	35.37 (30.49-38.78; 2.22)	35.64	1.02	1.28	1.65	0.33125
miRPlus-F1163	53.26 (43.58-60.3; 3.73)	53.88	49.84 (41.88-54.29; 2.67)	50.28	1.07	3.50	12.27	0.01097

miRPlus-F1180	66.81 (54.5-77.16; 5.34)	67.31	63.62 (55.24-85.92; 6.23)	61.73	1.05	1.84	3.40	0.16209
miRPlus-F1181	109.76 (27.38-244.94; 72.38)	82.77	114.76 (17.2-238.22; 50.85)	116.57	0.96	-0.27	0.07	0.84834
miRPlus-F1185	33.45 (27.92-56.18; 5.94)	32.98	36.02 (28.71-51.89; 5.08)	35.43	0.93	-1.56	2.43	0.23636
miRPlus-F1193	38.22 (35.15-42.3; 1.94)	38.11	38.08 (31.78-41.19; 2.38)	38.58	1.00	0.21	0.04	0.88123
miRPlus-F1194	60.03 (43.45-83.11; 8.28)	59.00	60.82 (48.24-93.75; 10.95)	58.35	0.99	-0.27	0.07	0.84834
miRPlus-F1195	39.76 (27.64-71.41; 9.83)	37.22	40.08 (23.79-51.7; 6.99)	40.22	0.99	-0.13	0.02	0.92430
miRPlus-F1206	52.22 (45.67-57.2; 2.93)	52.06	51.26 (44.73-59.86; 3.26)	50.93	1.02	1.02	1.04	0.44675
miRPlus-F1210	34.02 (29.24-36.59; 1.69)	34.12	32.74 (28.43-38.49; 2.2)	32.70	1.04	2.08	4.33	0.11428
miRPlus-F1215	55.17 (48.09-61.47; 2.91)	55.03	54.88 (50.41-66.58; 4.38)	53.43	1.01	0.26	0.07	0.85898
miRPlus-F1221	31.98 (28.13-35; 1.64)	31.69	32.11 (28.36-37.62; 1.91)	32.31	1.00	-0.23	0.05	0.87288
miRPlus-F1225	73.08 (55.15-98.41; 11.12)	73.37	69.36 (51.15-98.47; 9.11)	68.24	1.05	1.24	1.53	0.34983
miRPlus-F1237	49.5 (39.3-58.82; 4.85)	48.25	45.71 (39.76-59.83; 4.93)	45.10	1.08	2.59	6.71	0.05024
miRPlus-G1246-3p	32.14 (25.96-51.49; 5.91)	30.30	34.97 (28.04-41.33; 4.22)	35.30	0.92	-1.85	3.43	0.16082
			**Non-microRNA small RNAs**					
5S rRNA	2563.2 (1267.99-4282.75; 863.32)	2582.97	2422.39 (1101.1-4155.69; 905)	2233.34	1.06	0.54	0.29	0.68737
RNU1	1115.03 (723.8-1647.81; 247.94)	1079.05	874.65 (558.88-1322.21; 202.21)	891.35	1.27	3.60	12.93	0.00900
RNU5	750.37 (312.69-1551.95; 310.9)	707.52	521.01 (287.79-721.19; 111.97)	487.70	1.44	3.34	11.19	0.01474
RNU6-1	552.41 (200.64-957.1; 184.89)	524.22	390.61 (158.38-711.13; 118.08)	398.52	1.41	3.54	12.52	0.01011
RNU6-1/RNU6-2	4037.13 (1555.19-7285.9; 1552.97)	3512.91	2878.75 (1296.67-4590.25; 896.93)	2817.61	1.40	3.10	9.63	0.02172
SNORD10	395.28 (201.26-583.26; 106.43)	408.93	332.8 (202.68-439.39; 64.76)	331.16	1.19	2.41	5.80	0.07019
SNORD118	56.72 (37.59-110.16; 15.89)	53.12	49.08 (40.27-58.44; 6.02)	48.99	1.16	2.16	4.67	0.10175
SNORD12	36.67 (25.04-53.65; 6.86)	36.13	33.13 (23.36-40.5; 4.06)	33.81	1.11	2.12	4.48	0.10884
SNORD13	513.58 (251.4-958.23; 204.28)	513.76	384.05 (200.06-539.05; 88.93)	389.30	1.34	2.80	7.83	0.03604
SNORD14B	36.56 (30.13-44.46; 3.44)	35.94	34.79 (28.86-48.19; 4.46)	34.03	1.05	1.47	2.17	0.26306
SNORD2	277.69 (116.93-839.63; 156.92)	232.73	185.25 (122.98-252.06; 36.39)	189.34	1.50	2.77	7.67	0.03759
SNORD3@	602.26 (185.1-1339.42; 261.49)	573.19	415.53 (208.38-666.89; 104.4)	420.78	1.45	3.19	10.20	0.01896
SNORD38B	146.23 (74.81-330.15; 61.25)	141.87	116.07 (72.68-153.78; 20.48)	118.06	1.26	2.25	5.07	0.08952
SNORD44	132.65 (50.95-329.62; 65.03)	109.98	91.02 (61.4-127.72; 17.84)	91.17	1.46	2.98	8.87	0.02689
SNORD48	742.63 (272.3-1863.18; 364.75)	694.18	545.86 (290.51-801.48; 109.44)	550.54	1.36	2.49	6.21	0.06005
SNORD49A	109 (48.36-321.28; 56.01)	100.26	90.83 (66.53-125.57; 16.98)	86.58	1.20	1.50	2.24	0.25280
SNORD4A	112.29 (57.74-270.85; 49.75)	101.91	93.26 (50.88-130.23; 17.88)	97.18	1.20	1.73	3.01	0.18659
SNORD6	402.48 (150.99-872.54; 184.71)	381.96	334.08 (183.12-445.09; 70.67)	350.08	1.20	1.67	2.78	0.20322
SNORD65	702.76 (285.64-1351.94; 251.77)	716.24	593.72 (287.84-978.6; 190.64)	580.88	1.18	1.65	2.74	0.20600
SNORD68	128.87 (55.47-256.89; 49.39)	117.59	111.65 (66.48-160.16; 21.63)	112.32	1.15	1.54	2.36	0.24108

[a]RNAs are listed alphanumerically. Some probes recognize multiple species of RNAs and members of such sets of RNAs are listed together. Fold-change values are ratios of mean values for the case and control cohorts. Adjusted P values, calculated from moderated t-statistics, are q-values and indicate false discovery rates with the Bejamini-Hochberg method. Moderated t- and F-statistics were calculated using the limma Bioconductor package in R. RNAs differentially expressed between the case and control cohorts (adjusted P <0.05) are shown in bold.
