# Supplementary material for: MicroRNA Expression Profiles of Whole Blood in Lung Adenocarcinoma
Source: PLoS One. 2012 Sep 28;7(9):e46045. doi: 10.1371/journal.pone.0046045 (PMC3460960; doi:10.1371/journal.pone.0046045)
Supplement: Text S1 — R codes for processing of microarray data, differential gene expression analysis using moderated t-statistics in limma Bioconductor package, leave-one-out cross-validation analyses with top-scoring pairs (TSP) and linear support vector machines (SVM) classification methods, and Monte Carlo cross-validation analyses with TSP and SVM, are shown with annotation and information about the computing platform and R packages. (PDF) [file pone.0046045.s007.pdf]

Text S1

R codes for processing of microarray data, differential gene expression analysis using moderated t-statistics in limma Bioconductor package, leave-one-out cross-validation analyses with top-scoring pairs (TSP) and linear support vector machines (SVM) classification methods, and Monte Carlo cross-validation analyses with TSP and SVM, are shown with annotation and information about the computing platform and R packages.

1. Processing of microarray data

```
# Santosh Patnaik, 30 Jan 2011
# Mac OS X 10.6.6, R 2.12.1, Biobase 2.10.0, limma 3.6.9, reshape2 1.1, futile.matrix 1.0.1
library('limma')

# all files provided by Exiqon
targets <- readTargets('Samplesinfo.txt')
files <- targets[, c('Cy3', 'Cy5')]
# read-in flag-values, assigning a value of 0 if the Imagene flag-value is > 0, and a 1 if < 1
myWtFun <- function(exclude.flags=c(1, 2, 3, 4, 5, 6, 7)) function(obj) 1-(obj$Flag %in%
exclude.flags)
RG <- read.maimages(files, source='imagene', wt.fun=myWtFun(c(1, 2, 3, 4, 5, 6, 7)))
colnames(RG) <- targets$Name
RG$genes <- readGAL('gal_208300-A,208301-A,208302-A_lot33014-33014_hsa-and-related-
vira_from_mb160,miRPlus.gal')
RG$printer <- getLayout(RG$genes)

# background-correction using 'normexp' method with offset=10
RG <- backgroundCorrect(RG, method='normexp', offset=10)
# within-array normalization using 'global Loess' method with span=1/3 and flag-values weighed
MA <- normalizeWithinArrays(RG, weights=RG$weights, method='loess', span=1/3)
# exclude samples 9, 19 and 34 because of poor array-data quality, and sample 15 because of
metastases
MA <- MA[, -c(9, 15, 19, 34)]
# between-array normalization using 'Rquantile' method
MA <- normalizeBetweenArrays(MA, method='Rquantile')
RG <- new('RGList', RG.MA(MA))

# Hy3, Hy5 and Hy3/Hy5 values for probe-set summarization using mean if max/min < cutoffAgg;
else, median
cutoffAgg = 1.5
library(reshape2)
hy3 <- RG$G
hy5 <- RG$R
fc <- hy3/hy5
myAggFun <- function(x){ifelse(cutoffAgg<(max(x)/min(x)), median(x), mean(x))}
l<-list('hy3','hy5','fc')
for(i in 1:length(l)){
 dat <- get(l[[i]])
 rownames(dat) <- RG$genes$Name
 dat <- dcast(melt(dat), Var1~Var2, myAggFun, fill=NaN)
 rownames(dat) <- dat$Var1
 dat$Var1 <- NULL
 write.table(dat, file=paste(l[[i]], '.txt', sep=''), sep='\t', col.names=NA)
 assign(l[[i]], dat)
}

# filter for 'expressed' if more than a cutoffExpFrac of samples have Hy3 values > cutoffExpFold-
times 'Empty' probe value
cutoffExpFold = 3
cutoffExpFrac = 0.25
hy3RelEmpty <- apply(scale(hy3, center=FALSE, scale=t(hy3[c('Empty'), ])), 1:2,
function(x){ifelse(x > cutoffExpFold, 1, 0)})
rnaExp <- rownames(hy3RelEmpty[rowSums(hy3RelEmpty) > ncol(hy3RelEmpty)*cutoffExpFrac,])
hy3Exp <- hy3[rnaExp,]
fcExp <- fc[rnaExp,]
```

```
write.table(hy3Exp, file='hy3Exp.txt', sep='\t', col.names=NA)
write.table(fcExp, file='fcExp.txt', sep='\t', col.names=NA)

# filter for human 'expressed' RNAs or microRNAs; the pattern used to identify may not work with
another array version
patHsa <- c('(hsa-miR)|(hsa-let)|(5S)|(RNU)|(SNORD)')
patHsaMir <- c('(hsa-miR)|(hsa-let)')
library(futile.matrix)
write.table(select(hy3Exp, row.pat=patHsa), file='hy3ExpHsa.txt', sep='\t', col.names=NA)
write.table(select(fcExp, row.pat=patHsa), file='fcExpHsa.txt', sep='\t', col.names=NA)
write.table(select(hy3Exp, row.pat=patHsaMir), file='hy3ExpHsaMir.txt', sep='\t', col.names=NA)
write.table(select(fcExp, row.pat=patHsaMir), file='fcExpHsaMir.txt', sep='\t', col.names=NA)
```

2. Differential gene expression analysis using moderated t-statistics in limma

```
# Santosh Patnaik, 14 Feb 2011
# Mac OS X 10.6.6, R 2.12.1, Biobase 2.10.0, limma 3.6.9

library(Biobase)
library(limma)

# cohort membership; c for cancer, h for healthy; cannot use numbers
classes <- factor(c('c', 'c', 'c', 'c', 'c', 'c', 'c', 'c', 'c', 'c', 'c', 'c', 'c', 'c', 'c',
'c', 'c', 'c', 'c', 'c', 'c', 'c', 'h', 'h', 'h', 'h', 'h', 'h', 'h', 'h', 'h', 'h', 'h', 'h',
'h', 'h', 'h', 'h', 'h', 'h', 'h', 'h', 'h', 'h', 'h'))

design <- model.matrix(~0 + classes)
colnames(design) <- levels(classes)
contrast <- makeContrasts(c-h, levels=design)

exp <- readExpressionSet(exprsFile='hy3ExpHsaMir.txt', sep='\t', row.names=1, header=T)
fit <- lmFit(exp, design)
fit2 <- contrasts.fit(fit, contrast)
fit2 <- eBayes(fit2)
# all genes with Benj.-Hoch.-adj. P < 0.05, arranged by logFC which is NOT log(fold-change)
# topTable(fit2, adjust='BH', number=Inf, p.value=0.05)
write.fit(fit2, file='limmaDiffHy3ExpHsaMir.txt', sep='\t', adjust='BH')
```

3. Leave-one-out cross-validation analyses with TSP and SVM classification methods

```
# Santosh Patnaik, 3 Feb 2011
# Mac OS X 10.6.6, R 2.12.1, Biobase 2.10.0, tspair 1.8.0, CMA 1.8.1, limma 3.6.9, WilcoxCV 1.0.2

# 'exp' is the expression table, a matrix; 'classes' is list of known classes
exp <- as.matrix(read.table(file='hy3ExpHsaMir.txt', sep='\t', row.names=1, header=T,
check.names=F))
# best is to use 0 and 1 as class labels (instead of 'p', 'g', etc.) with 1 for 'positive' items
(positive for recurrence, or for disease, etc.)
classes <- c(1, 1, 1, 1, 1, 1, 1, 1, 1, 1, 1, 1, 1, 1, 1, 1, 1, 1, 1, 1, 1, 1, 0, 0, 0, 0, 0, 0,
0, 0, 0, 0, 0, 0, 0, 0, 0, 0, 0, 0, 0, 0, 0, 0, 0)
yesPredVal = 1 # class label for 'positive' items in 'classes'

# TSP LOOCV
library(tspair)
res <- matrix(nrow=0, ncol=4)
colnames(res) <- c('iteration', 'score', 'gene1', 'gene2')
tp <- tn <- fp <- fn <- 0
out = 'TSP LOOCV:\nSample\tClass label\tPredicted class label\n'

for(i in 1:ncol(exp)){
 # actual prediction work
 tsp <- tspcalc(exp[, -i], classes[-i])
 pred <- predict(tsp, exp)[i]
 known <- classes[i]
 if(pred == known){
  if(pred == yesPredVal){tp <- tp+1}
  else{tn <- tn+1}
```

```
 }else{
  if(pred == yesPredVal){fp <- fp+1}
  else{fn <- fn+1}
 }
 out = paste(out, colnames(exp)[i], '\t', known, '\t', pred, '\n', sep='')
 res <- rbind(res, c(i, tsp$tspscore[1], rownames(exp)[tsp$index[1, 1]],
rownames(exp)[tsp$index[1, 2]]))
}

# output
out = paste(out, ncol(exp), ' predictions; accuracy, sensitivity, specificity, PPV and NPV in
fractions are ', round((tp+tn)/(tp+tn+fp+fn), 3), ', ', round(tp/(tp+fn), 3) , ', ',
round(tn/(tn+fp), 3), ', ', round(tp/(tp+fp), 3) , ', and ', round(tn/(tn+fn), 3), ',
respectively; mean (range; SD) of TSP scores for the topmost TS pairs is ',
round(mean(as.numeric(res[, 'score'])), 2), ' (', round(min(as.numeric(res[, 'score'])), 2), '-',
round(max(as.numeric(res[, 'score'])), 2), '; ', round(sd(as.numeric(res[, 'score'])), 2), ').
There are a total of ', length(unique(res[, 'gene1'])), ' and ', length(unique(res[, 'gene2'])),
' unique genes for gene-1 and -2, respectively, in ', ncol(exp), ' topmost TS pairs with
following occurrence frequencies in fractions:\n', sep='')
tspGeneTab <- as.matrix(append(res[, 'gene1'], res[, 'gene2']))
tspUnqGene <- unique(tspGeneTab)
for(i in 1:length(tspUnqGene)){
 out = paste(out, tspUnqGene[i], round(length(tspGeneTab[tspGeneTab[, 1]==tspUnqGene[i],
])/ncol(exp), 3), '\n', sep='\t')
}
cat(out)

# SVM LOOCV
library(CMA)
diffTest = 'limma'
diffGeneNum = 15
svmCost <- c(0.1, 0.2, 0.5, 1, 2, 5, 10, 20, 50)
tp <- tn <- fp <- fn <- 0

# actual prediction work
lsets <- GenerateLearningsets(n=ncol(exp), y=classes, method=c('LOOCV'))
svm <- classification(t(exp), factor(classes), learningsets=lsets,
genesellist=list(method=diffTest), classifier=svmCMA, nbgene= diffGeneNum,
tuninglist=list(grids=list(cost=svmCost)), probability=T, models=T)
svm <- join(svm)

# output
# CMA internally re-names class labels
out = paste('SVM LOOCV with ', diffTest, ' and nbgene=', diffGeneNum, ':\nSample\tClass
label\tClass label as per CMA\tPredicted class label as per CMA\n', sep='')
for(i in 1:ncol(exp)){
 # since this is the way LOOCV training sets are generated in CMA
 j <- ncol(exp)-(i-1)
 pred <- svm@yhat[j]
 known <- svm@y[j]
 if(pred == known){
  if(pred == yesPredVal){tp <- tp+1}
  else{tn <- tn+1}
 }else{
  if(pred == yesPredVal){fp <- fp+1}
  else{fn <- fn+1}
 }
 out = paste(out, colnames(exp)[i], '\t', classes[i], '\t', known, '\t', pred, '\n', sep='')
}
out = paste(out, ncol(exp), ' predictions; accuracy, sensitivity, specificity, PPV and NPV in
fractions are ', round((tp+tn)/(tp+tn+fp+fn), 3), ', ', round(tp/(tp+fn), 3) , ', ',
round(tn/(tn+fp), 3), ', ', round(tp/(tp+fp), 3) , ', and ', round(tn/(tn+fn), 3), ',
respectively. ', sep='')
svmGenes <- GeneSelection(t(exp), classes, learningsets=lsets, method=diffTest)
temp <- numeric()
for(i in 1:ncol(exp)){
 temp <- c(temp, toplist(svmGenes, k=diffGeneNum, iter=i, show=F)$index)
}
temp2 <- unique(temp)
```

```
out = paste(out, ' A total of ', length(temp2), ' genes occur in the ', ncol(exp), ' classifiers,
with occurrence frequencies in fractions of:\n', sep='')
for(i in 1:length(temp2)){
 out = paste(out, rownames(exp)[temp2[i]], '\t', round(sum(temp==temp2[i])/ncol(exp), 3), '\n',
sep='')
}
cat(out);
```

4. Monte Carlo cross-validation analyses with TSP and SVM classification methods

```
# Santosh Patnaik, 16 Feb 2011
# Mac OS X 10.6.6, R 2.12.1, Biobase 2.10.0, tspair 1.8.0, CMA 1.8.1, limma 3.6.9, WilcoxCV 1.0.2

# 'exp' is the expression table, a matrix; 'classes' is list of known classes
exp <- as.matrix(read.table(file='hy3ExpHsaMir.txt', sep='\t', row.names=1, header=T,
check.names=F))
# best is to use 0 and 1 as class labels (instead of 'p', 'g', etc.) with 1 for 'positive' items
(positive for recurrence, or for disease, etc.)
classes <- c(1, 1, 1, 1, 1, 1, 1, 1, 1, 1, 1, 1, 1, 1, 1, 1, 1, 1, 1, 1, 1, 1, 0, 0, 0, 0, 0, 0,
0, 0, 0, 0, 0, 0, 0, 0, 0, 0, 0, 0, 0, 0, 0, 0)
yesPredVal = 1 # class label for 'positive' items in 'classes'

### Begin TSP MCCV
library(tspair)
library(WilcoxCV)
myNumFun <- function(x, y){round(y(as.numeric(x), na.rm=T), 4)}
set.seed(631)
out = ''
out2 = '\nEffect of varying the training-set size:\nTraining-set size\tSuccessful
iterations\tMean acc.\tSD acc.\tMean sens.\tSD sens.\tMean spec.\tSD spec.\tMean PPV\tSD
PPV\tMean NPV\tSD NPV\tTotal genes in the classifiers\n'

niter = 1000

for(g in 0:8){
 ntest=3+g*3
 result <- matrix(nrow=0, ncol=10)
 colnames(result) <- c('trainSetSize', 'iteration', 'acc', 'sens', 'spec', 'ppv', 'npv', 'score',
'gene1', 'gene2')
 diffGenesAll <- c()

 # generate 2*niter training/test sets but use niter sets (valid TSP calc. requires min. 2
classes)
 set <- generate.split(niter=2*niter, n=ncol(exp), ntest=ntest)

 actualIters = 0
 for(h in 1:(2*niter)){
  if(actualIters > niter-1){
   break;
  }
  # actual prediction work
  trExp <- exp[, -set[h, ]]
  tsExp <- exp[, set[h, ]]
  trClasses <- classes[-set[h, ]]
  tsClasses <- classes[set[h, ]]
  if(1 < length(unique(trClasses))){
   actualIters = actualIters+1
   tsp <- tspcalc(trExp, trClasses)
   tp <- tn <- fp <- fn <- 0
   for(i in 1:ncol(tsExp)){
    pred <- predict(tsp, tsExp)[i]
    known <- tsClasses[i]
    if(pred == known){
     if(pred == yesPredVal){tp <- tp+1}
     else{tn <- tn+1}
    }else{
     if(pred == yesPredVal){fp <- fp+1}
     else{fn <- fn+1}
```

```
    }
   }
   result <- rbind(result, c(ncol(exp)-ntest, h, (tp+tn)/(tp+tn+fp+fn), tp/(tp+fn), tn/(tn+fp),
tp/(tp+fp), tn/(tn+fn), tsp$tspscore[1], rownames(exp)[tsp$index[1, 1]],
rownames(exp)[tsp$index[1, 2]]))
   diffGenesAll <- c(diffGenesAll, rownames(exp)[tsp$index[1, 1]], rownames(exp)[tsp$index[1,
2]])
  } # end if valid TSP
 } # end for h

 # output accuracy, etc.
 out = paste(out, '\nTSP MCCV using ',  niter, ' attempted iterations and ', actualIters, '
successful iterations, with ', ncol(exp)-ntest, ' of ', ncol(exp), ' total samples used for
training:\nThe means (ranges; SDs) of prediction accuracy, sensitivity, specificity, PPV and NPV
in fractions are ',
 myNumFun(result[, 'acc'], mean), ' (', myNumFun(result[, 'acc'], min), '-', myNumFun(result[,
'acc'], max), '; ', myNumFun(result[, 'acc'], sd), '), ',
 myNumFun(result[, 'sens'], mean), ' (', myNumFun(result[, 'sens'], min), '-', myNumFun(result[,
'sens'], max), '; ', myNumFun(result[, 'sens'], sd), '), ',
 myNumFun(result[, 'spec'], mean), ' (', myNumFun(result[, 'spec'], min), '-', myNumFun(result[,
'spec'], max), '; ', myNumFun(result[, 'spec'], sd), '), ',
 myNumFun(result[, 'ppv'], mean), ' (', myNumFun(result[, 'ppv'], min), '-', myNumFun(result[,
'ppv'], max), '; ', myNumFun(result[, 'ppv'], sd), '), and ',
 myNumFun(result[, 'npv'], mean), ' (', myNumFun(result[, 'npv'], min), '-', myNumFun(result[,
'npv'], max), '; ', myNumFun(result[, 'npv'], sd), '), respectively. The mean (range; SD) of the
topmost TSP scores is ',  myNumFun(result[, 'score'], mean), ' (', myNumFun(result[, 'score'],
min), '-', myNumFun(result[, 'score'], max), '; ', myNumFun(result[, 'score'], sd), '). There are
a total of ', length(unique(result['gene1'])), ' and ', length(unique(result['gene2'])), ' unique
genes for gene-1 and -2, respectively, in the ', actualIters, ' topmost TS pairs with following
occurrence frequencies, in fractions:\n', sep='')

 diffGenesAllUnq <- unique(diffGenesAll)
 for(i in 1:length(diffGenesAllUnq)){
  out = paste(out, diffGenesAllUnq[i], round(sum(diffGenesAll == diffGenesAllUnq[i])/actualIters,
4), '\n', sep='\t')
 }

 # output split-size effect
 out2 = paste(out2, ncol(exp)-ntest, '\t', actualIters, '\t', myNumFun(result[, 'acc'], mean),
'\t', myNumFun(result[, 'acc'], sd), '\t', myNumFun(result[, 'sens'], mean), '\t',
myNumFun(result[, 'sens'], sd), '\t', myNumFun(result[, 'spec'], mean), '\t', myNumFun(result[,
'spec'], sd), '\t', myNumFun(result[, 'ppv'], mean), '\t', myNumFun(result[, 'ppv'], sd), '\t',
myNumFun(result[, 'npv'], mean), '\t', myNumFun(result[, 'npv'], sd), '\t',
length(diffGenesAllUnq), '\n', sep='')
} # end for g

cat(out, out2, sep='')
### End TSP MCCV

### Begin SVM MCCV
library(CMA)
myNumFun <- function(x, y){round(y(as.numeric(x), na.rm=T), 4)}
set.seed(631)
out = ''
out2 = '\nEffect of varying the training-set size:\nTraining-set size\tSuccessful
iterations\tMean acc.\tSD acc.\tMean sens.\tSD sens.\tMean spec.\tSD spec.\tMean PPV\tSD
PPV\tMean NPV\tSD NPV\tTotal genes in the classifiers\n'

niter = 1000
diffTest = 'limma'
diffGeneNum = 15
svmCost <- c(0.1, 0.2, 0.5, 1, 2, 5, 10, 20, 50)

for(g in 0:8){
 ntest = 3+g*3
 result <- matrix(nrow=0, ncol=7)
 colnames(result) <- c('trainSetSize', 'iteration', 'acc', 'sens', 'spec', 'ppv', 'npv')
 diffGenes <- numeric()
```

```
 # generate learning-sets with 2*niter members in case some have to be removed; valid SVM
classification requires min. 2 classes; urther, inner loop validation requires certain number of
members of each class in ach training set
 lsets <- GenerateLearningsets(n=ncol(exp), y=classes, method=c('MCCV'), niter=2*niter,
ntrain=ncol(exp)-ntest)
 temp <- lsets@learnmatrix
 for(i in 1:(2*niter)){
  if(2 > length(unique(classes[lsets@learnmatrix[i, ]]))){
   temp <- lsets@learnmatrix[-i, ]
  }
 }
 lsets@learnmatrix <- temp[1:niter, ]
 lsets@iter <- niter

 # genes in classifiers
 svmGenes <- GeneSelection(t(exp), classes, learningsets=lsets, method=diffTest)
 svmTune <- tune(t(exp), factor(classes), learningsets=lsets, genesel=svmGenes,
classifier=svmCMA, nbgene=diffGeneNum, grids=list(cost=svmCost))
 # actual prediction work
 svm <- classification(t(exp), factor(classes), learningsets=lsets, genesel=svmGenes,
classifier=svmCMA, nbgene=diffGeneNum, tuneres=svmTune, probability=T, models=T)
 svm <- join(svm)

 actualIters=0
 for(h in 1:niter){
  m <- ntest*(h-1)
  #
  if(1 < length(unique(classes[-lsets@learnmatrix[h,]]))){
   actualIters = actualIters+1
   tp <- tn <- fp <- fn <- 0
   for(i in 1:ntest){
    pred <- svm@yhat[m+i]
    known <- svm@y[m+i]
    if(pred == known){
     if(pred == yesPredVal){tp <- tp+1}
     else{tn <- tn+1}
    }else{
     if(pred == yesPredVal){fp <- fp+1}
     else{fn <- fn+1}
    }
   }
   result <- rbind(result, c(ncol(exp)-ntest, h, (tp+tn)/(tp+tn+fp+fn), tp/(tp+fn), tn/(tn+fp),
tp/(tp+fp), tn/(tn+fn)))
   diffGenes <- c(diffGenes, toplist(svmGenes, k=diffGeneNum, iter=h, show=F)$index)
  } # end if valid SVM
 } # end for h

 # output accuracy, etc.
 out = paste(out, 'SVM MCCV using ',  niter, ' attempted iterations and ', actualIters, '
successful iterations, with ', ncol(exp)-ntest, ' of ', ncol(exp), ' total samples used for
training:\nThe means (ranges; SDs) of prediction accuracy, sensitivity, specificity, PPV and NPV
in fractions are ',
myNumFun(result[, 'acc'],mean), ' (', myNumFun(result[, 'acc'], min), '-', myNumFun(result[,
'acc'], max), '; ', myNumFun(result[, 'acc'], sd), '), ',
 myNumFun(result[, 'sens'], mean), ' (', myNumFun(result[, 'sens'], min), '-', myNumFun(result[,
'sens'], max), '; ', myNumFun(result[, 'sens'], sd), '), ',
 myNumFun(result[, 'spec'], mean), ' (', myNumFun(result[, 'spec'], min), '-', myNumFun(result[,
'spec'], max), '; ', myNumFun(result[, 'spec'], sd), '), ',
 myNumFun(result[, 'ppv'], mean), ' (', myNumFun(result[, 'ppv'], min), '-', myNumFun(result[,
'ppv'], max), '; ', myNumFun(result[, 'ppv'], sd), '), and ',
 myNumFun(result[, 'npv'], mean), ' (', myNumFun(result[, 'npv'], min), '-', myNumFun(result[,
'npv'], max), '; ', myNumFun(result[, 'npv'], sd), '), respectively.\n', sep='')

 # output classifier genes
 diffGenesUnq <- unique(diffGenes)
 out = paste(out, 'A total of ', length(diffGenesUnq), ' genes occur in the ', actualIters, '
classifiers, with occurrence frequencies in fractions:\n', sep='')
 for(i in 1:length(diffGenesUnq)){
```

```
  out = paste(out, rownames(exp)[diffGenesUnq[i]], '\t', round(sum(diffGenes ==
diffGenesUnq[i])/actualIters, 3), '\n', sep='')
 }

 # output split-size effect
 out2 = paste(out2, ncol(exp)-ntest, '\t', actualIters, '\t', myNumFun(result[, 'acc'], mean),
'\t', myNumFun(result[, 'acc'], sd), '\t', myNumFun(result[, 'sens'], mean), '\t',
myNumFun(result[, 'sens'], sd), '\t', myNumFun(result[, 'spec'], mean), '\t', myNumFun(result[,
'spec'], sd), '\t', myNumFun(result[, 'ppv'], mean), '\t', myNumFun(result[, 'ppv'], sd),
'\t', myNumFun(result[, 'npv'], mean), '\t', myNumFun(result[, 'npv'], sd), '\t',
length(diffGenesUnq), '\n', sep='')
} # end for g

cat(out, out2, sep='')
### End SVM MCCV
```
